# Mental, behavioral and neurodevelopmental disorders in the ICD-11: an international perspective on key changes and controversies

**DOI:** 10.1186/s12916-020-1495-2

**Published:** 2020-01-27

**Authors:** Dan J. Stein, Peter Szatmari, Wolfgang Gaebel, Michael Berk, Eduard Vieta, Mario Maj, Ymkje Anna de Vries, Annelieke M. Roest, Peter de Jonge, Andreas Maercker, Chris R. Brewin, Kathleen M. Pike, Carlos M. Grilo, Naomi A. Fineberg, Peer Briken, Peggy T. Cohen-Kettenis, Geoffrey M. Reed

**Affiliations:** 1 SA Medical Research Council Unit on Risk & Resilience in Mental Disorders, Dept of Psychiatry & Neuroscience Institute, University of Cape Town, Cape Town, South Africa; 2 Centre for Addiction and Mental Health, Hospital for Sick Children, University of Toronto, Toronto, ON Canada; 3 Department of Psychiatry and Psychotherapy, Medical Faculty, Heinrich-Heine University, Düsseldorf, Germany; 4 Deakin University, IMPACT, the Institute for Mental and Physical Health and Clinical Translation, School of Medicine, Barwon Health, Geelong, Australia; 5 Orygen, The National Centre of Excellence in Youth Mental Health and the Centre for Youth Mental Health, Parkville, Australia; 6 Florey Institute for Neuroscience and Mental Health, Parkville, Australia; 7 Department of Psychiatry, University of Melbourne, Parkville, Australia; 8 Bipolar Disorders Unit, Hospital Clinic, Institute of Neurosciences, University of Barcelona, IDIBAPS, CIBERSAM, Barcelona, Catalonia Spain; 9 Department of Psychiatry, University of Campania ‘L. Vanvitelli’, Naples, Italy; 10 Department of Developmental Psychology, Interdisciplinary Center Psychopathology and Emotion Regulation, University of Groningen, Groningen, The Netherlands; 11 Department of Psychology – Psychopathology and Clinical Intervention, University of Zurich, Zurich, Switzerland; 12 Research Deparment of Clinical, Educational and Health Psychology, University College London, London, UK; 13 Department of Psychiatry, Columbia University Vagelos College of Physicians and Surgeons, New York, NY USA; 14 Department of Psychiatry, Yale University School of Medicine, New Haven, CT USA; 15 Hertfordshire Partnership University NHS Foundation Trust and University of Hertfordshire, Welwyn Garden City, UK; 16 Institute for Sex Research, Sexual Medicine & Forensic Psychiatry, University Medical Centre Hamburg-Eppendorf, Hamburg, Germany; 17 Department of Medical Psychology, Amsterdam UMC, Amsterdam, The Netherlands; 18 Department of Mental Health and Substance Abuse, World Health Organization, Geneva, Switzerland

**Keywords:** Mental disorder, Diagnosis, Classification, ICD-11

## Abstract

An update of the chapter on Mental, Behavioral and Neurodevelopmental Disorders in the International Classification of Diseases and Related Health Problems (ICD) is of great interest around the world. The recent approval of the 11th Revision of the ICD (ICD-11) by the World Health Organization (WHO) raises broad questions about the status of nosology of mental disorders as a whole as well as more focused questions regarding changes to the diagnostic guidelines for specific conditions and the implications of these changes for practice and research. This Forum brings together a broad range of experts to reflect on key changes and controversies in the ICD-11 classification of mental disorders. Taken together, there is consensus that the WHO’s focus on global applicability and clinical utility in developing the diagnostic guidelines for this chapter will maximize the likelihood that it will be adopted by mental health professionals and administrators. This focus is also expected to enhance the application of the guidelines in non-specialist settings and their usefulness for scaling up evidence-based interventions. The new mental disorders classification in ICD-11 and its accompanying diagnostic guidelines therefore represent an important, albeit iterative, advance for the field.

## Introduction

### Dan J. Stein (Fig. 1) and Geoffrey M. Reed (Fig. 2)

Classification systems for mental disorders continue to receive considerable attention. Work by the World Health Organization (WHO) on the Eleventh Revision of the International Classification of Diseases and Related Health Problems (ICD-11) and by the American Psychiatric Association on the Fifth Edition of the Diagnostic and Statistical Manual of Mental Disorders (DSM-5) has led to vigorous debates in the scientific literature, among clinicians and health advocates, and in the lay media (for example, regarding the inclusion of gaming disorder and compulsive sexual behaviour disorder) [1, 2]. In the context of the recent approval of the ICD-11 by the World Health Assembly and its pending implementation around the world, a number of questions arise regarding the status of nosology of mental disorders as a whole as well as about changes to the diagnostic guidelines for specific conditions and the implications of these changes for practice and research.

**Fig. 1:**
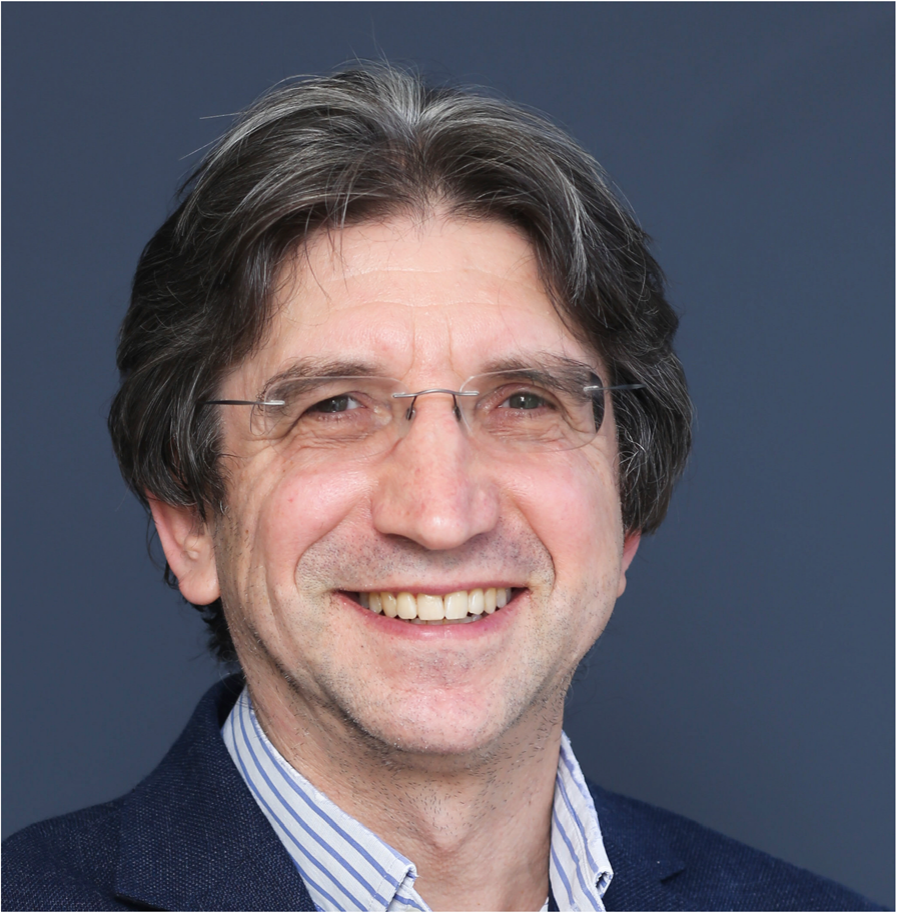
Dan Stein. Dr. Dan J. Stein is Professor and Chair of the Dept of Psychiatry at the University of Cape Town, Director of the South African Medical Research Council’s Unit on Risk & Resilience in Mental Disorders, and Scientific Director of UCT’s Neuroscience Institute. His training includes doctoral degrees in both clinical neuroscience and philosophy, and a post-doctoral fellowship in psychopharmacology. He is a clinician-scientist-advocate whose work has long focused on anxiety and related disorders, including obsessive–compulsive spectrum conditions and post-traumatic stress disorder, as well as other issues relevant to the African context

**Fig. 2:**
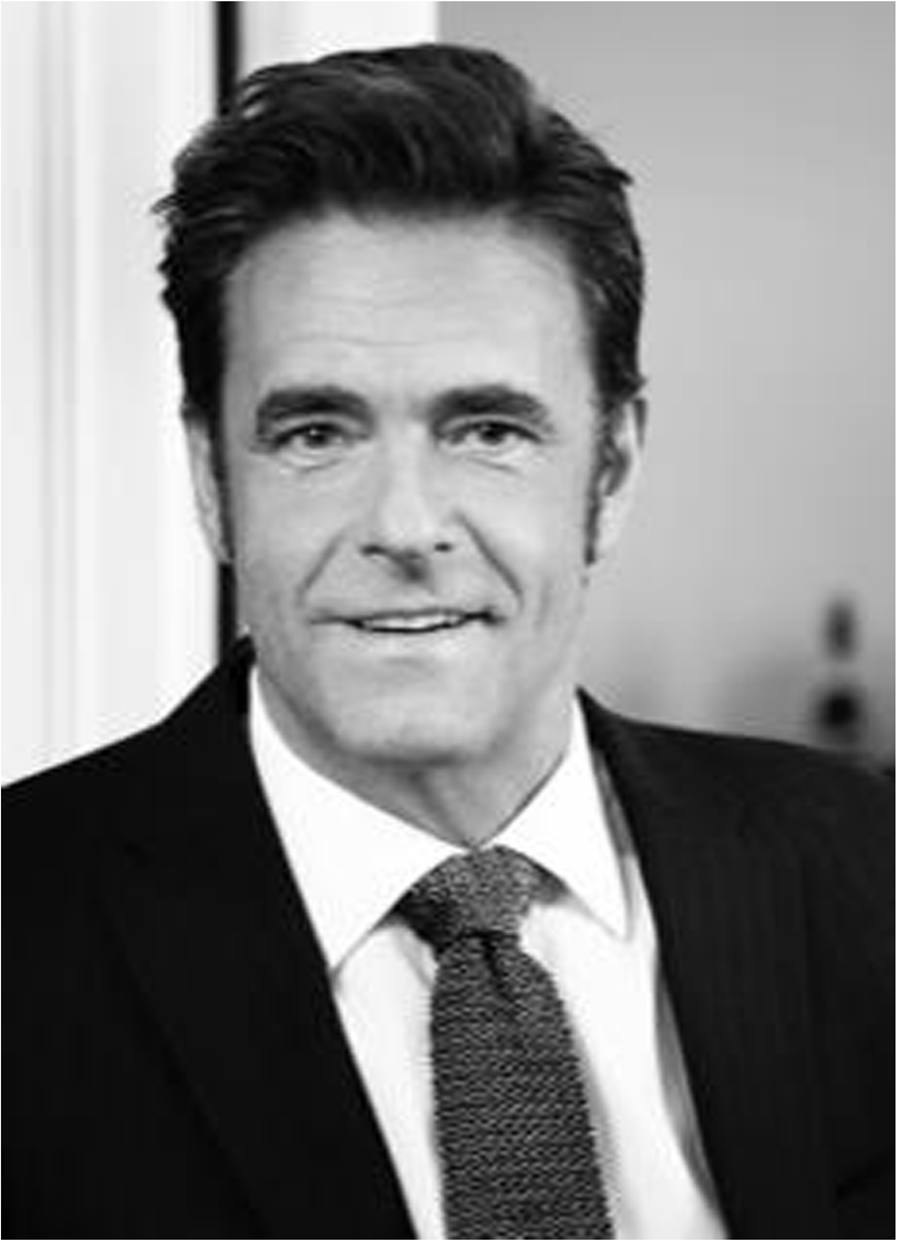
Geoffrey Reed. Dr. Geoffrey M. Reed has coordinated the development of ICD-11 classification of Mental, Behavioral or Neurodevelopmental Disorders in the Department of Mental Health and Substance Abuse, World Health Organization since 2008. He is Professor of Medical Psychology, Global Mental Health Programs, Department of Psychiatry, Columbia University Vagelos College of Physicians and Surgeons. He founded the WHO Global Clinical Practice Network (http://gcp.network), comprising more than 15,000 mental health and primary care professionals from 158 countries contributing directly to ICD-11 through participation in field studies

First, the need for two classification systems — the ICD and the DSM — has been questioned. At first glance, it seems odd that there should be contrasting approaches to mental disorders. However, different diagnostic systems are arguably needed for different purposes. The DSM-III, for example, made an enormous contribution by ensuring that diagnostic procedures were reliable, providing a foundation for mental health research and leading to DSM criteria being rapidly adopted by investigators. During the development of the chapter on Mental, Behavioral or Neurodevelopmental Disorders in ICD-11, a particular focus was placed on clinical utility and global applicability [3] based on the idea that this would lead to a classification system that is of particular value for global mental health, providing WHO member states and health professionals with a better tool for reducing the mental health treatment gap and the global burden of mental disorders.

Second, does the updating of classification systems in fact strengthen mental health practice and research? A great deal of dissatisfaction has been expressed with current nosologies; criticism ranges from the view that our nosologies have medicalized problems of daily life, to the view that the constructs employed in existing classifications are insufficiently grounded in contemporary neuroscience. Still, it is difficult to argue with the general principle that comprehensive evaluation and differential diagnosis is a key part of clinical practice. Furthermore, there have been impressive advances in the science of nosology — a classification system that is more reliable, with better diagnostic validity and greater clinical utility should certainly contribute to stronger practice and research.

Third, how important is the recent release of the ICD-11, with its updated chapter on Mental, Behavioral or Neurodevelopmental disorders? What specific changes does it contribute to psychiatric nosology and how valuable are these changes for clinicians and patients?

In order to begin to address these questions, we asked a range of authors to comment on revisions to the ICD-11 from the perspective of their specific areas of expertise. The commentaries that follow cover a range of important mental disorders and will bring readers up to date on many of the questions and controversies regarding their diagnosis, on how the ICD-11 has addressed these, and the implications for clinical practice and research.

The focus of the commentaries in this article is the Clinical Descriptions and Diagnostic Guidelines (CDDG) for ICD-11 Mental, Behavioual and Neurodevelopmental Disorders developed by the WHO Department of Mental Health and Substance Abuse. The version of the ICD-11 intended as a basis for statistical reporting does not provide sufficient information for reliable clinical application, The CDDG is a more comprehensive version that provides clinicians with detailed clinical guidance for diagnosing mental disorders in clinical settings. A draft review version of the CDDG for most disorder groupings is available for review and comment by members of the Global Clinical Practice Network (https://gcp.network). The statistical version is available at https://icd.who.int/dev11/l-m/en.

## Neurodevelopmental disorders in the ICD-11: has the term outlived its usefulness?

### Peter Szatmari (Fig. 3)

Classification systems are made to carry a heavy burden. They serve multiple purposes in supporting clinical activities, treatment planning, conducting research and in policy decision-making. In child and youth mental health, and in particular neurodevelopmental disorders, diagnosis and classification also tend to generate an enormous amount of controversy, which can confuse stakeholders who rely on consensus-based classification systems to make policy and clinical decisions. The Neurodevelopmental Disorders section of the ICD-11 represents a significant departure from the ICD-10 and is very much aligned with recent decisions made by the DSM-5. But it is still hard to please everybody.

**Fig. 3:**
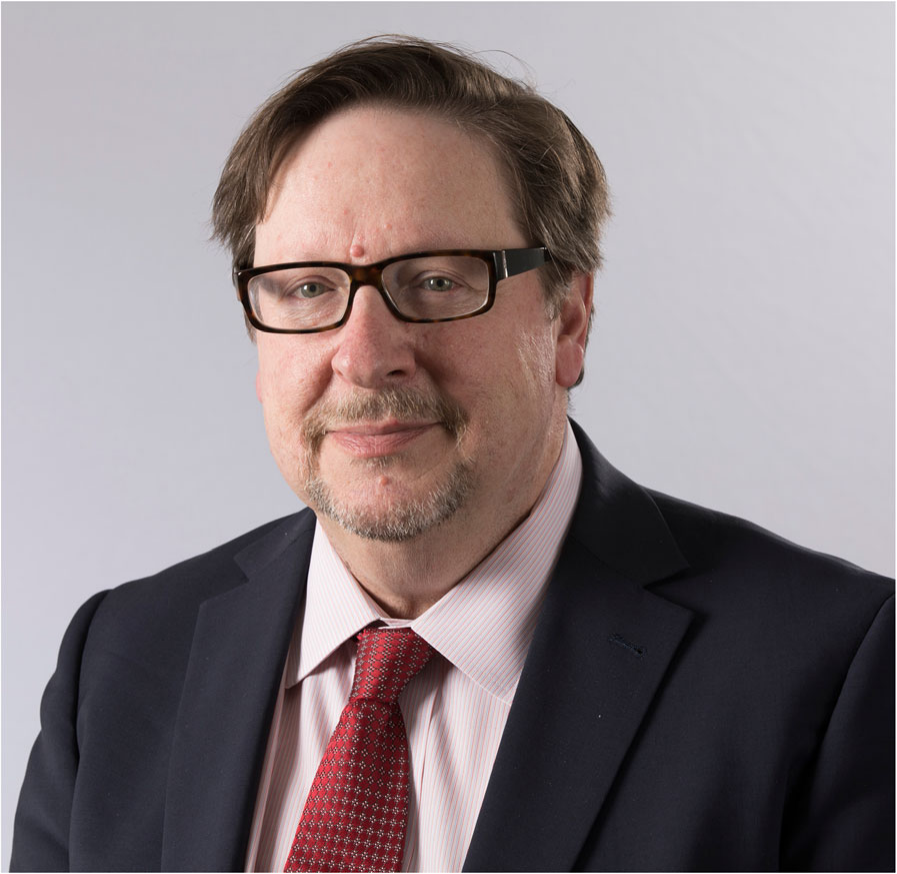
Peter Szatmari. Dr. Szatmari is the Chief of the Child and Youth Mental Health Collaborative at the Hospital for Sick Children, the Centre for Addiction and Mental Health, and the University of Toronto in Toronto, Canada. He has worked in the area of autism spectrum disorders for many years focusing on diagnosis and classification, genetics, and follow-up studies

The term ‘neurodevelopmental disorders’ has a long history, yet it had not been included in previous editions of the ICD or the DSM. The term applies to a group of disorders of early onset that affect both cognitive and social communicative development, are multi-factorial in origin, display important sex differences where males are more commonly affected than females, and have a chronic course with impairment generally lasting into adulthood [4]. The term distinguishes these disorders from other more common disorders of childhood, such as anxiety and mood disorders, which were thought to arise from some type of psychosocial adversity and have a more episodic course. In the ICD-11, the category ‘neurodevelopmental disorders’ includes (1) disorders of intellectual development, (2) developmental speech or language disorders, (3) autism spectrum disorders (ASD), (4) developmental learning disorders, (5) developmental motor coordination disorder, (6) attention deficit hyperactivity disorder (ADHD), (7) stereotyped movement disorder, and (8) a remainder category labeled ‘other neurodevelopmental disorders’.

There are a number of very important departures from the ICD-10, which are consistent with recent literature and follow, in spirit, the changes from the DSM-IV to the DSM-5 [5]. First, the ICD-10 does not have a specific grouping for neurodevelopmental disorders and uses slightly different terminology for the specific conditions that have been included within it — ‘mental retardation’, ‘disorders of psychological development’, and ‘pervasive developmental disorder’ are the terms used instead. Second, hyperkinetic disorder (now termed ‘attention deficit hyperactivity disorder’ in ICD-11) appears under the ICD-10 category of ‘behavioral and emotional disorders with onset in childhood or adolescence’. Third, it is notable that, in the ICD-10, pervasive developmental disorder is exclusionary for hyperkinetic disorder, a stipulation that is no longer present in the ICD-11. Now, in the ICD-11, both ASD and ADHD may co-exist in the same individual. The age of onset for ASD is now in the early developmental period rather than being specified as having an onset by 3 years of age.

Other major changes include the fact that the eight different pervasive developmental disorders in the ICD-10, including childhood autism, atypical autism and Asperger syndrome, have disappeared entirely and are now grouped together under one category, namely ASD. This is a notable change that still arouses some controversy [6]. Several systematic reviews have found that the distinctions between these subtypes appear to be of dubious diagnostic validity or to represent quantitative rather than qualitative variation [7, 8]. In both the DSM-5 and the ICD-11, grouping all these individuals together is now accompanied by adding different ‘specifiers’ to the ASD diagnosis in an attempt to take account of the enormous heterogeneity inherent in the disorder’s presentation. These specifiers include intellectual level, language level, medical or genetic comorbidities, and mental health comorbidities.

While there is general support for ‘lumping’ the ASDs rather than ‘splitting’ them, there has been little or no research on the clinical utility of these specifiers nor on whether these are the ‘right’ specifiers. It is to be hoped that this conceptualization of a single disorder with multiple specifiers will foster a new generation of studies that attempts to consider the remarkable heterogeneity seen in ASD both between individuals with ASD but also within the same person with ASD over time.

The recognition that ADHD and ASD can coexist is also an important refinement that is extremely useful since there is good evidence that ASD individuals with concurrent ADHD can benefit from stimulant medications [9]. There is also growing evidence that ASD and ADHD share common genetic variants, similar psychological deficits and neuroimaging differences [10–12].

Nevertheless, despite the term ‘neurodevelopmental disorders’ now being official, it could be argued that the designation has outlived its usefulness — the various conditions contained under this grouping differ from each other (from severe ASD to mild coordination disorder) such that they have little in common. Therefore, the allocation of treatment interventions and prognosis cannot be generalized from one neurodevelopmental disorder to another. If clinical utility is the prime criterion for the added value of diagnostic terms, then ‘neurodevelopmental disorders’ as a meta-term appears to make a minimal contribution.

Moreover, it could also be argued that all disorders with onset in childhood or adolescence are neurodevelopmental disorders. Schizophrenia, mood (including bipolar), and anxiety disorders are all brain-based disorders. They have also, on occasion, been referred to as neurodevelopmental disorders, especially schizophrenia [13, 14], as they involve difficulties in the execution of intellectual, motor, language, or social functions as well as other domains that arise from alterations in brain circuits. Similar to the definition of neurodevelopmental disorders in the ICD-11, the presumptive etiology of mood disorders in childhood and adolescence, for example, is also ‘complex’ and is thought to arise from ‘physical’ processes (inflammatory processes, chronic sleep disturbance, possibly the microbiome) and genetic factors [15–17] as well as from various types of stressful life events. The growing awareness of the comorbidity of mood and anxiety disorders with various neurodevelopmental disorders (once the children reach adolescence) is another indication that the boundary between neurodevelopmental and non-neurodevelopmental disorders in the ICD-11 is ambiguous.

In other words, what does not constitute a neurodevelopmental disorder among disorders that arise in childhood and adolescence? More importantly, what is the clinical utility of grouping them together and separating them from disruptive behaviour and internalizing disorders? It is possible that mood and anxiety disorders are more closely associated with psychosocial adversity than with neurodevelopmental disorders; however, surely these are quantitative rather than qualitative differences. Furthermore, so many evidence gaps remain in our understanding of etiology and pathogenesis that to build the foundation of a classification system on unknown and assuredly complex aetiological factors is a fragile enterprise. The term represents child and adolescent psychiatry’s version of the old ‘functional’ versus ‘organic’ distinction that has long been done away with in adult psychiatry following remarkable advances in neuroscience. Perhaps it is time to put the term ‘neurodevelopmental disorders’ into the history books as well?

## Schizophrenia or other primary psychotic disorders

### Wolfgang Gaebel (Fig. 4)

The development of the chapter on Mental, Behavioral or Neurodevelopmental Disorders in the ICD-11 included collaboration with stakeholders; consideration of applications in clinical practice, research, teaching and training, health statistics, and public health; and a focus on clinical utility, global applicability, and reduction of disease burden [18]. The initial proposals of the WHO Working Group on the Classification of Psychotic Disorders for the ICD-11 [19], which I chaired and comprised experts from all global regions, were revised in response to public comment, expert peer review and results from field testing under the guidance of an international advisory group appointed by the WHO Department of Mental Health and Substance Abuse.

**Fig. 4:**
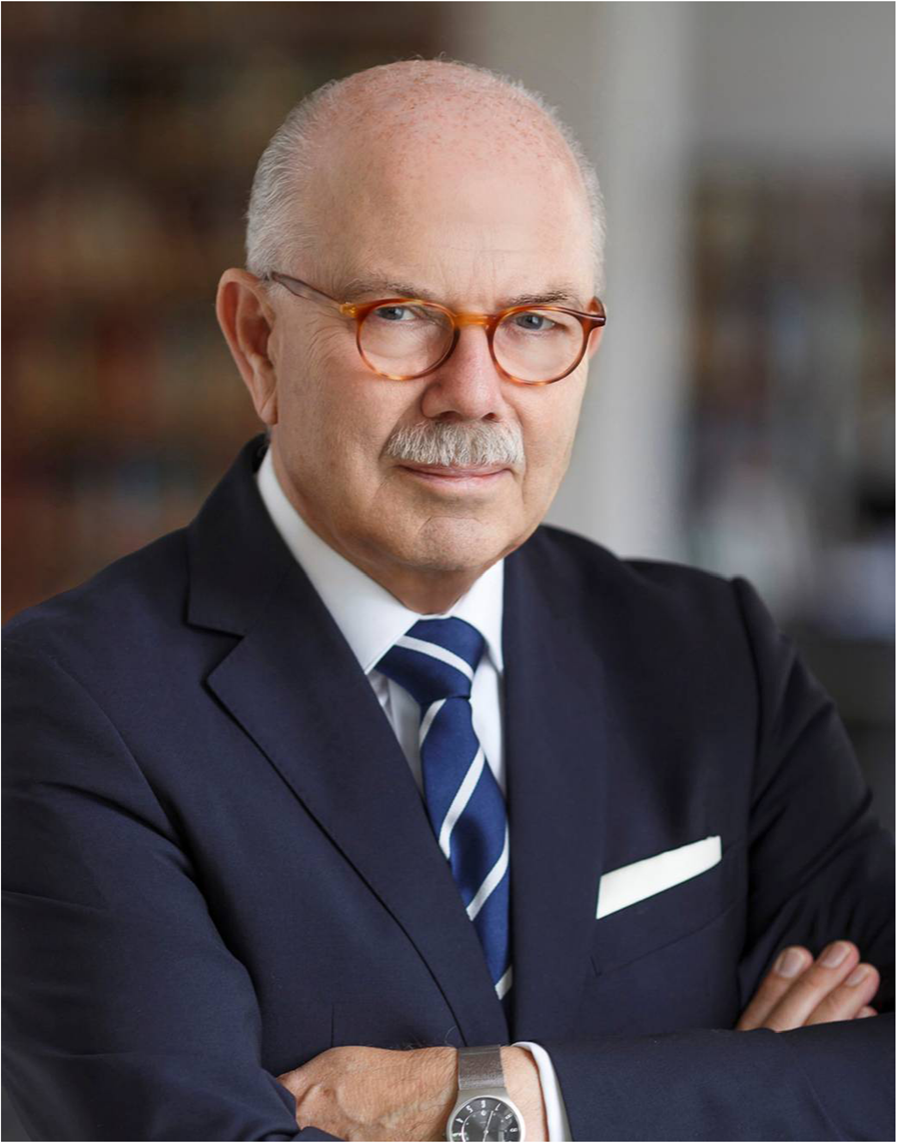
Wolfgang Gaebel. Dr. med. Wolfgang Gaebel is Professor of Psychiatry and Psychotherapy at the Heinrich-Heine University Düsseldorf, Germany. He was Director of the Department of Psychiatry and Psychotherapy and Head of the LVR-Klinikum Düsseldorf (LVR-KD) from 1992 until 2016. From 2014 to 2016, he was also Founding Director of the LVR-Institute for Mental Healthcare Research (LVR-IVF). Since 2014, he is also Head of the WHO Collaborating Centre on Quality Management and Empowerment in Mental Health at the LVR-KD

The most important changes in the classification of psychotic disorders from the ICD-10 to ICD-11, based on evidence review and consensus of the Working Group, include the following:

- The ICD-10 section entitled ‘Schizophrenia, schizotypal and delusional disorders’ has been replaced by ‘Schizophrenia or other primary psychotic disorders’. The term ‘primary’ was intended to distinguish these disorders from bipolar and other mental or medical disorders that may include psychotic symptoms.
- Accordingly, non-primary (i.e. ‘secondary’) psychotic disorders, such as psychotic disorders due to substance use or withdrawal and psychotic disorders in general medical conditions, are respectively placed in the sections of the mental disorders chapter corresponding to ‘Disorders due to substance use’ and ‘Mental and behavioural disorders associated with disorders or diseases classified elsewhere’. However, the unique features of the ICD-11, including its fully relational and electronic development for complex coding, make it possible to cross-list substance-induced psychotic disorders and those associated with general medical conditions in the section on primary psychotic disorders as well, enhancing clinical utility while still retaining the ability to allocate and aggregate these disorders appropriately for public health reporting.
- The overall structure proposed for the ICD-11 block on ‘Schizophrenia or other primary psychotic disorders’ is as follows:
  - ◦ Schizophrenia
  - ◦ Schizoaffective disorder
  - ◦ Acute and transient psychotic disorder (ATPD)
  - ◦ Schizotypal disorder
  - ◦ Delusional disorder
  - ◦ Other primary psychotic disorders
  - ◦ Unspecified primary psychotic disorders
- Disorders in this section continue to be categorized on the basis of their psychopathological profile, duration, or course characteristics, as described in the Clinical Descriptions and Diagnostic Guidelines being developed for use by mental health professionals in clinical settings [20].
- Similar to the DSM-5, in the ICD-11, the nine ICD-10 schizophrenia subtypes (paranoid, hebephrenic, catatonic, etc.) are now omitted because of their longitudinal instability and lack of prognostic validity [21], and have been replaced by a system of coded symptom and course qualifiers (see below). Although first-rank symptoms are somewhat deemphasized [22], a diagnosis of schizophrenia requires the presence of at least two of seven symptom categories, including at least one ‘core’ symptom. Core symptoms include delusions, hallucinations, experiences of influence, passivity or control, and disorganized thinking. Symptoms should have been clearly present for most of the time during a period of at least 1 month, hence retaining the ICD-10 duration requirement. If the symptom requirements for schizophrenia are fulfilled but the duration is less than 1 month, ‘Other specified primary psychotic disorder’ would be the appropriate diagnosis until the duration requirement is met.
- In the ICD-11, a diagnosis of schizoaffective disorder should be made only when the symptom criteria of schizophrenia and of a moderate or severe mood episode are fulfilled simultaneously or within a few days of each other. The total duration requirement is 1 month, including both mood and schizophrenic symptoms. According to long-term studies, 10% of persons with schizoaffective disorder have only a single episode, while decades-long, symptom-free intervals may occur between episodes among people who have had more than one episode [23, 24].
- For the category of ‘acute and transient psychotic disorder’ (ATPD), the ICD-11 places the diagnostic focus on sudden onset, brief duration and high variability/fluctuation of psychotic and affective symptoms (i.e. the ‘polymorphic’ clinical presentation). For simplification and due to a lack of empirical support for the prognostic and therapeutic relevance of the distinctions made in the ICD-10 among several subtypes of ATPD, only F23.0 ‘Acute polymorphic psychotic disorder without symptoms of schizophrenia’ is retained as the core clinical category for ATPD, whereas F23.3 ‘Other acute predominantly delusional disorder’ is incorporated into the revised category delusional disorder. F23.1 ‘Acute polymorphic psychotic disorder with symptoms of schizophrenia’ and F23.2 ‘Acute schizophrenia-like psychotic disorder’ are replaced by ‘Other specified primary psychotic disorder’ if the duration is less than 1 month, and should be diagnosed as schizophrenia if all diagnostic requirements are met.
- ICD-10 categories F22 ‘Persistent delusional disorder’, F23.3 ‘Other acute predominantly delusional psychotic disorder’, and F24 ‘Induced delusional disorder’, which is a very rare entity, are collapsed into a single diagnostic category of ‘Delusional disorder’, omitting the durational qualifier ‘persistent’ to adapt to its durational heterogeneity and also to simplify the classification system.
- Schizotypal disorder remains largely unchanged in the ICD-11. As in the ICD-10, it is considered a validated member of the schizophrenia ‘spectrum’ as a potential precursor or subsyndromal variant of schizophrenia [25] rather than a personality disorder and is therefore included in the section on primary psychotic disorders.
- As an alternative to subtypes, coded qualifiers to describe the course of the disorder as well as symptom presentation are included in the section and can be applied to all primary psychotic disorders. Course qualifiers allow the differentiation of first- and multiple-episode cases, and between acute episodes with symptoms, full or partial remission, and a chronic course due to different prognostic implications [26]. Symptom qualifiers include the presence of positive, negative, depressive, manic, psychomotor, and cognitive symptoms, each of which may be rated as mild, moderate, or severe. A qualifier for cognitive symptoms is intended to provide more diagnostic and therapeutic attention to cognitive symptoms as these are linked to functional outcome [27]. The ‘psychomotor symptoms’ qualifier includes catatonic symptoms. In addition, catatonia is also included as a separate category in the ICD-11 [28, 29].

The inclusion of mental and behavioral disorders alongside all the other diagnostic medical entities in healthcare is an important feature of the ICD that has consequences for clinical practice and research. A classification that uses a common framework across all disease and disorder areas is more likely to be used by all specialties and general healthcare workers in a similar way, thereby yielding comparable health statistics results. The advanced classification and coding framework of the ICD-11 will also facilitate research in fields of epidemiology to analyze mechanisms of comorbidity, causal relationships, and treatment options. Another opportunity is the provision of conceptual parity of psychopathology with the rest of the medical system for clinical, administrative, and financial functions in healthcare [18]. As the ICD-11 will be used worldwide by a large range of health professionals [30], its definitions and diagnostic guidelines should not only be reliable (and valid), but also useful and easy to implement by different users in different clinical settings and around the world.

As internet-based and clinical field trials have demonstrated for psychotic disorders, diagnostic reliabilities for the ICD-11 compared to the ICD-10 have markedly improved for most of the diagnostic categories in this section [31]. Improvements have also been shown for judgments on the ease of use and related utility measures based on the use of diagnostic guidelines by health professionals and brief descriptions by medical records coders [32], although reliabilities for the latter show room for improvement. Accordingly, the introduction of the ICD-11 in the field of psychotic disorders will contribute to improved mental health care, particularly due to a more dimensional approach to symptoms characterization that allows for more individualized treatment selection. More generally, implementation of the ICD-11 chapter on mental disorders will profit from intense education and training of the mental health workforce.

## Bipolar disorders

### Michael Berk (Fig. 5) and Eduard Vieta (Fig. 6)

The ICD-11 brings significant changes from the ICD-10. Concordant with the overarching strategy, most changes go towards harmonizing the ICD-11 with the DSM-5. Noting the imperfection of both systems, inevitable given the absence of a valid pathophysiological foundation, this is desirable, as diagnostic labels principally represent a common global language. Perhaps the greatest point of difference is that the DSM-5 retains the requirement for a set number of phenomena across diagnoses, while the ICD-11 offers a descriptive approach and allows the clinician to pattern-fit the diagnosis. Arguably, the DSM system is most useful for research, where objective, reproducible, and verifiable diagnoses are essential, but the ICD better captures the way most clinicians actually think and behave [33].

**Fig. 5:**
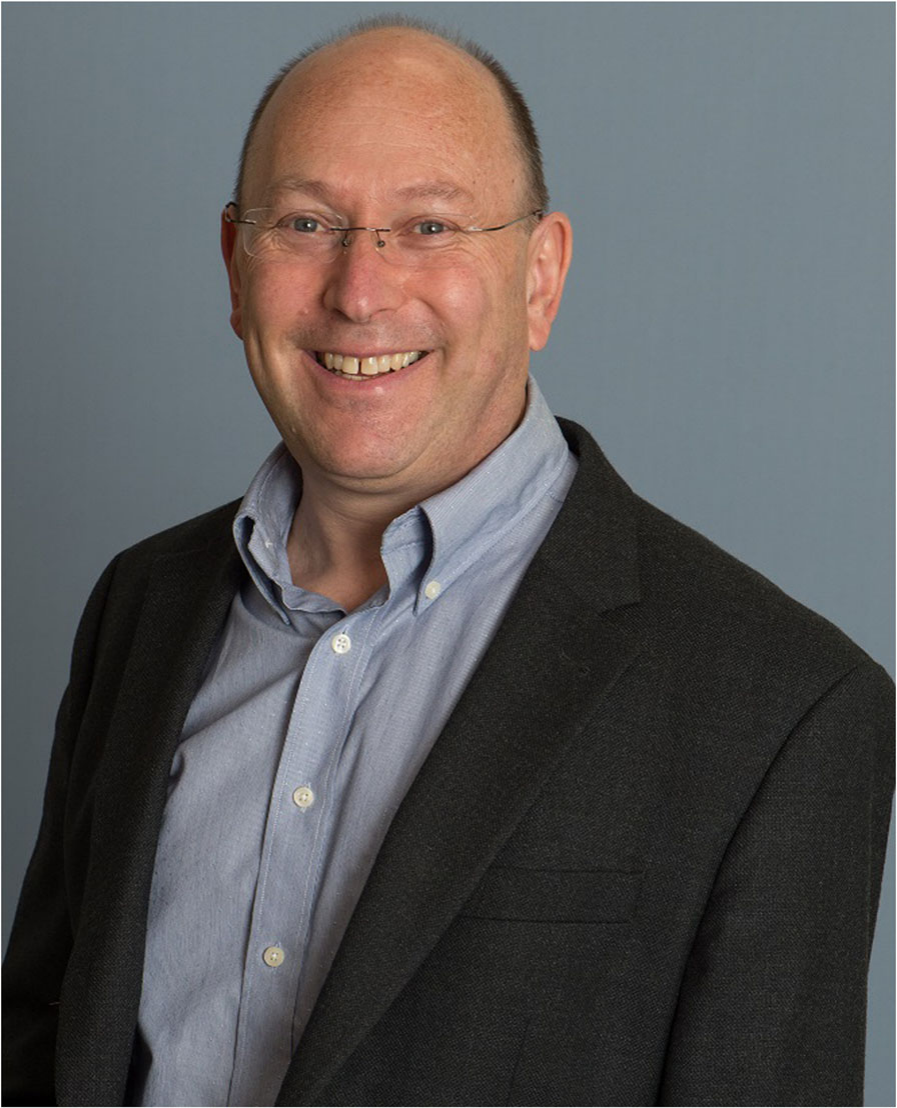
Michael Berk. Professor Michael Berk is currently a NHMRC Senior Principal research Fellow and is Alfred Deakin Chair of Psychiatry at Deakin University and Barwon Health, where he heads IMPACT, the Institute for Mental and Physical Health and Clinical Translation. He also is an Honorary Professorial Research fellow in the Department of Psychiatry, the Florey Institute for Neuroscience and Mental Health and Orygen Youth Health at Melbourne University, as well as in the School of Public Health and Preventive Medicine at Monash University. His major interests are in the discovery and implementation of novel therapies as well as risk factors and prevention of psychiatric disorders

**Fig. 6:**
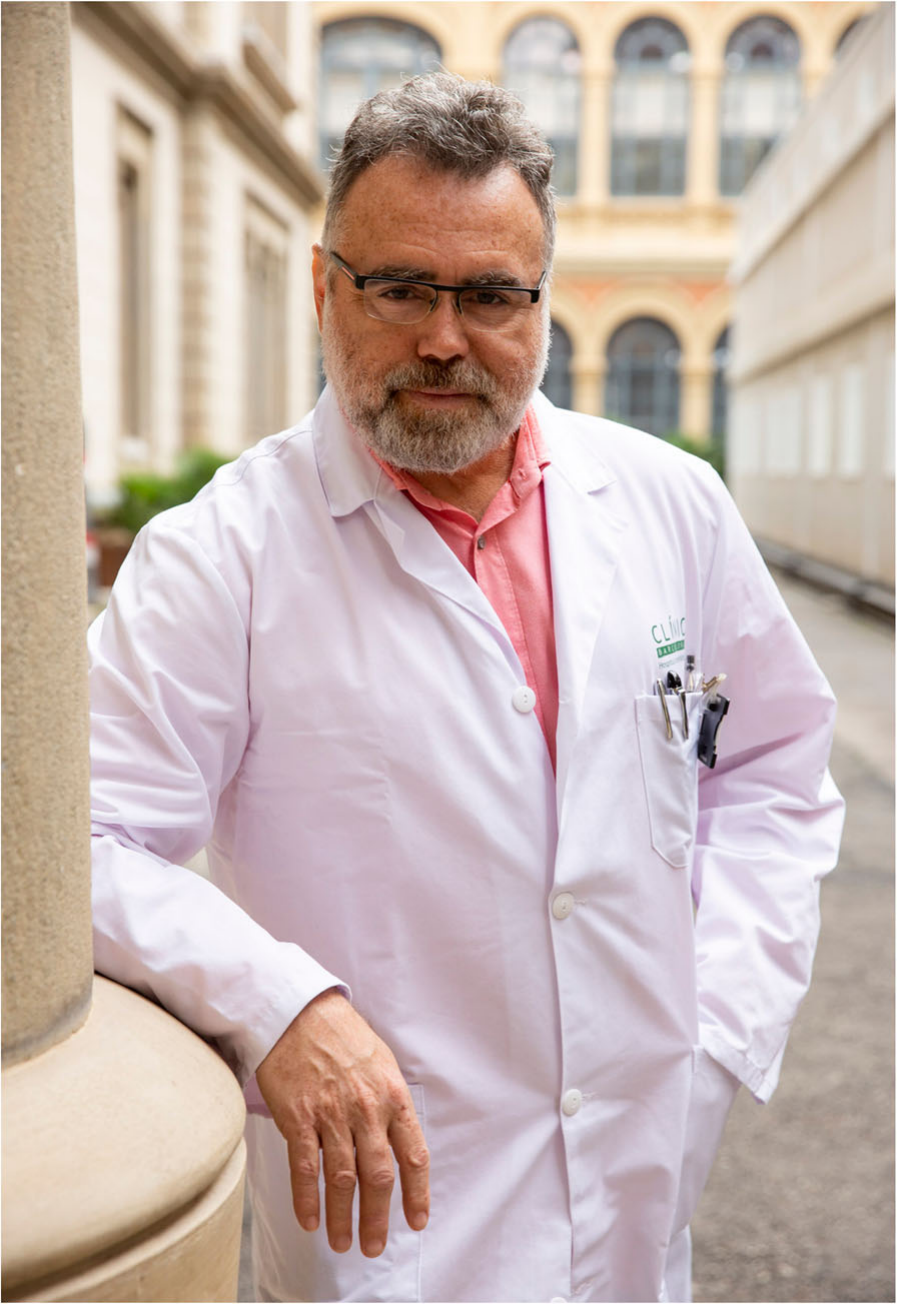
Eduard Vieta. Dr. Eduard Vieta is Professor of Psychiatry at the University of Barcelona, Chair of the Department of Psychiatry and Psychology at the Hospital Clinic, and Scientific Director of the Spanish Biomedical Research Network on Mental Health (CIBERSAM)

The ICD-11 considers bipolar disorders as mood disorders, as the ICD-10 did (but not the DSM-5, which lists them in a separate chapter). Perhaps the most significant change is that the ICD-10 required two or more episodes of elevated mood, whereas the ICD-11 has lowered the threshold to one or more manic or mixed episodes to make a diagnosis of bipolar I disorder. A manic episode is therefore no longer an independently diagnosable condition as it was in the ICD-10. This parallels a significant shift in clinical thinking. Orthodoxy had it that individuals should not start mood stabilizers until a couple of manic episodes had occurred. More recent evidence suggests that the response to treatment is best after the first episode [34] and declines thereafter, and this decline is associated with a potentially neuroprogressive process with substantial clinical and functional sequelae [35, 36]. Additionally, the ICD-11 now requires a duration of at least 1 week for mania, while the ICD-10 did not have duration criteria and the ICD-11 waives this if treatment is present.

Allowing a mixed episode is a further significant change from the ICD-10, which specified the requirement of hypomania or mania and depression. Again, mixed episodes and their treatment implications are increasingly recognized in the clinical literature [37] and this is a welcome change suggesting the need for a differentially tailored treatment approach. Mixed episodes are defined as being characterized by rapid alternation between or an admixture of prominent manic or depressive symptoms with a duration of at least 2 weeks, and therefore they remain in the ICD-11 as an episode type as opposed to the DSM-5, which converted those into a specifier. Rapid cycling is defined based on the frequency of episodes of mood disturbance, requiring at least four episodes in the past 12 months, as traditionally stated.

In the ICD-11, the diagnostic guideline for mania allows for euphoria, irritability, or expansiveness together with increased activity or subjectively increased energy as well as other characteristic manic symptoms, without specifying the number of symptoms. There remains controversy around the inclusion of irritability, which is a far more non-specific phenomenon with much overlap with disorders such as ADHD, conduct disorder, and personality disorders [38]. Allowing mania based principally in the context of irritability risks blurring the boundary between these disorders [39]. However, the requirement for increased activity or energy is an important and positive change, concordant with recent data suggesting a critical role of a biphasic change in mitochondrial energy generation as core to the biology of the disorder [40].

The ICD-10 described hypomania and included bipolar II disorder under the heading of ‘Other bipolar affective disorders’. The ICD-11 harmonizes this with the DSM system allowing a bipolar type II diagnosis with equal status to bipolar I disorder. In contrast to bipolar I disorder, where impulsive or reckless behaviour are part of the description, hypomania in bipolar II is defined as not causing marked impairment in function. The controversy around the duration of hypomania is avoided by defining the duration as lasting for at least several days.

Similarly, cyclothymia, which stood in the ICD-10 under a separate grouping, is now incorporated in the core bipolar or related disorders heading. These changes substantially harmonize the ICD-11 and DSM-5 systems, one overarching goal of the revision. Cyclothymia includes a duration definition of at least 2 years and requires that numerous hypomanic or depressive symptoms be present the majority of the time. While the hypomanic symptomatology may or may not meet threshold criteria for hypomania, but not mania, depression cannot be severe or prolonged enough to meet the diagnostic requirement for a depressive episode. Curiously, the cyclothymia rubric includes cycloid and cyclothymic personality. This is a significant change echoing the old Research Diagnostic Criteria that set the grounds for the DSM-III and earlier literature on the effect of temperament, suggesting a bridge between the core bipolar and unipolar mood disorders and the effect of mood instability so commonly seen in borderline and other personality disorders [41]. Finally, akin to the specifiers in the DSM system, the ICD-11 allows the use of qualifiers to refine the description of current mood episodes, including prominent anxiety, melancholy (in depressive episodes), current perinatal episode, seasonal patterns, and rapid cycling, but no mention is made of predominant polarity, a specifier with therapeutic implications under consideration for future editions of the DSM [42].

Overall, the harmonization of the ICD-11 with the DSM-5 is desirable, hopefully presaging greater global uniformity in the use of a critical diagnostic language and hence translation to evidence-based care. The weaknesses of the two systems are both debatable and unavoidable [43, 44] given the lack of any objective pathophysiological compass and the limited specificity of most currently available biomarkers in mental illness [45]. This is a welcome fillip to global clinical care [46].

## Depression

### Mario Maj (Fig. 7)

Depression is reported to be the most common mental disorder in the general population and one of the most important contributors to the global burden of disease worldwide. Therefore, its valid and reliable diagnosis is essential not only from the psychiatric perspective but also more generally with regards to public health.

**Fig. 7:**
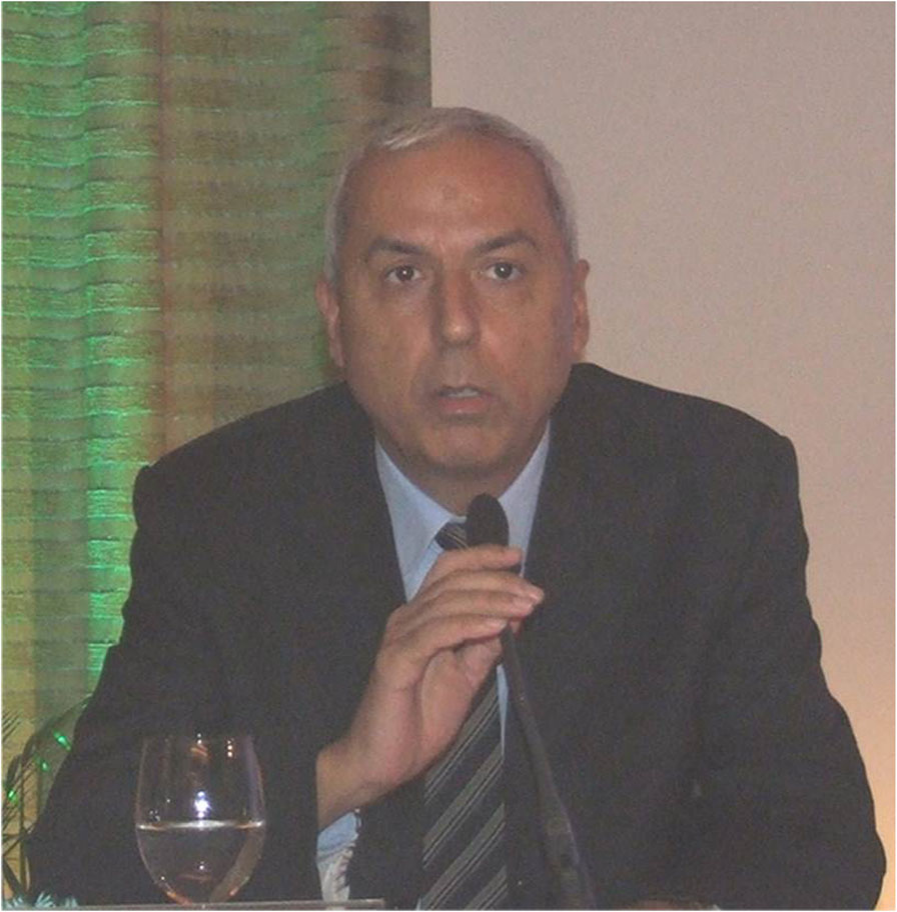
Mario Maj. Dr. Mario Maj is Professor of Psychiatry and Chairman at the Department of Psychiatry of the University of Campania L. Vanvitelli in Naples, Italy. He has been President of the European Psychiatric Association (2003–2004) and of the World Psychiatric Association (2008–2011). He is the Editor of World Psychiatry, the psychiatric journal with the highest impact factor (34.024). He has been a member of the International Advisory Group and Chairman of the Working Group on Mood and Anxiety Disorders for the ICD-11. He has been a member of the Working Group on Mood Disorders for the DSM-5

In the ICD-11, a depressive episode is defined by the concurrent presence of at least five out of a list of ten symptoms, which must occur most of the day, nearly every day, for at least 2 weeks. One of these symptoms must be depressed mood or markedly diminished interest or pleasure in activities. The mood disturbance must result in significant functional impairment and not be a manifestation of another health condition, due to the effects of a substance or medication, or better accounted for by bereavement.

The ten symptoms are depressed mood, markedly diminished interest or pleasure in activities, reduced ability to concentrate and sustain attention or marked indecisiveness, beliefs of low self-worth or excessive or inappropriate guilt, hopelessness about the future, recurrent thoughts of death or suicidal ideation or evidence of attempted suicide, significantly disrupted sleep or excessive sleep, significant changes in appetite or weight, psychomotor agitation or retardation, and reduced energy or fatigue. The list includes one symptom (hopelessness) that is not present in the DSM-5 criteria for major depression, but which was found to perform more strongly than approximately half of the DSM symptoms in differentiating depressive from non-depressive subjects [47].

The description of a depressive episode is one of the few instances in the ICD-11 in which specific thresholds are provided with respect to both number and duration of symptoms. Furthermore, the threshold concerning the number of symptoms (at least five) is made consistent with the DSM, whereas it was not in the ICD-10 (at least four).

A major difference between the ICD-11 and the DSM-5 relates to the so-called ‘bereavement exclusion’ — present in the DSM-IV but deleted in the DSM-5. In the ICD-11, as in the DSM-IV, the diagnosis of depression is not excluded in a person who is bereaved, but the threshold for that diagnosis is just made higher (as ordinarily happens in clinical practice) by requiring a longer duration of the depressive state (at least 1 month) and the presence of some symptoms that are unlikely to occur in ‘normal’ grief (extreme beliefs of low self-worth and guilt not related to the lost loved one, presence of psychotic symptoms, suicidal ideation, or psychomotor retardation). The ICD-11 approach to the bereavement issue has been supported (and the DSM-5 approach has been disproved) by longitudinal prospective studies documenting that the risk of subsequent depressive episodes during a period of follow-up was significantly lower in people with baseline bereavement-related versus non-bereavement-related major depression, and not different in the bereaved group than among controls without a history of depressive episodes [48, 49].

Interestingly, the ICD-11 states that a depressive episode is differentiated from a normal reaction to adverse life events (e.g. divorce, job loss) “*by the severity, range and duration of symptoms*” (as stated in the forthcoming WHO Clinical Descriptions and Diagnostic Guidelines). On the contrary, in the DSM-5, the decision of whether a response to a significant loss qualifies or not for a diagnosis of a major depressive episode is left, in a specific note, to ‘clinical judgment’, which contradicts the declared aim of that diagnostic system to overcome the “*vagueness and subjectivity inherent in the traditional diagnostic process*” [50].

In the ICD-11, the strategy to introduce ‘qualifiers’ (corresponding to the DSM-5 ‘specifiers’) to represent the heterogeneity of depression is adopted for the first time (no qualifiers were present in the ICD-10). The qualifiers proposed for depression are similar to the DSM-5 specifiers, with the exception that the DSM-5 specifier ‘with mixed features’ is absent in the ICD-11. In fact, the category ‘mixed episode’, eliminated in the DSM-5, is retained in the ICD-11; indeed, the DSM-5 definition of major depression with mixed features is highly controversial, as it includes typical manic symptoms (such as elevated mood and grandiosity) that have been found to be extremely rare among patients with mixed depression, while excluding symptoms (such as irritability, psychomotor agitation, and distractibility) that are frequently reported in mixed depression. Not surprisingly, major depression with mixed features, as defined in the DSM-5, has very different correlates in terms of treatment response compared to mixed depression as described in the literature [51].

The qualifier ‘with prominent anxiety symptoms’, introduced in the ICD-11, is of special clinical interest. Indeed, the presence of a significant anxiety component in a depressive episode is associated with a higher suicide risk, a longer duration of illness and a greater likelihood of non-response to treatment.

The ICD-11 approach to the assessment of the severity of the current depressive episode is analogous to that of the DSM-5, except that the number of depressive symptoms is not considered among the criteria (a depressive episode is regarded as mild, moderate, or severe depending on the intensity of the depressive symptoms and the degree of functional impairment). The characterization of the severity of depression remains unsatisfactory in both diagnostic systems, and the need for the inclusion of a simple and reliable rating scale for this purpose will have to be considered in the future.

A long and detailed section, missing in the ICD-10, is devoted in the ICD-11 to the delineation of the boundaries of depression with other mental disorders as well as with ‘normality’ and normal grief.

The ICD-11 guidelines for depression have been found to have substantial inter-clinician reliability and very good clinical utility in field trials [31, 52]. Therefore, it is expected that they will be well received in ordinary clinical practice worldwide.

## Anxiety disorders and obsessive–compulsive disorder

### Ymkje Anna de Vries (Fig. 8), Annelieke M. Roest (Fig. 9), Peter de Jonge (Fig. 10)

Anxiety disorders form a heterogeneous group defined by the presence of anxiety states such as worry, fear, or panic. They are characterized by a chronic course [53, 54] and an early age of onset [55]. Obsessive–compulsive disorder (OCD) shares some of these characteristics and has previously been considered an anxiety disorder (e.g. in the DSM-IV). We therefore discuss it alongside anxiety disorders.

**Fig. 8:**
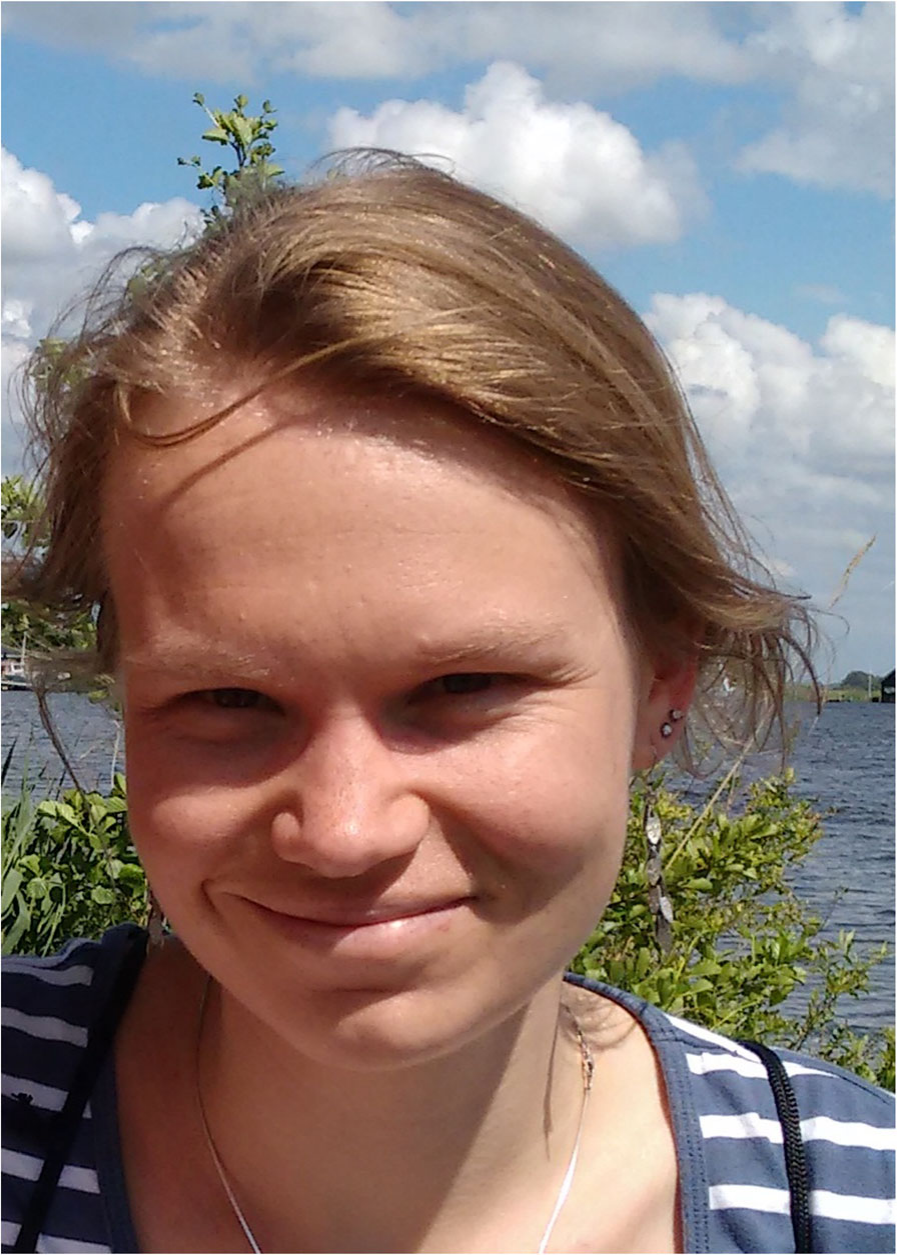
Ymkje Anna de Vries. Dr. Ymkje Anna de Vries is a postdoctoral researcher in the Developmental Psychology research group at the University of Groningen, the Netherlands. Her research is focused on the development and treatment of depressive and anxiety disorders

**Fig. 9:**
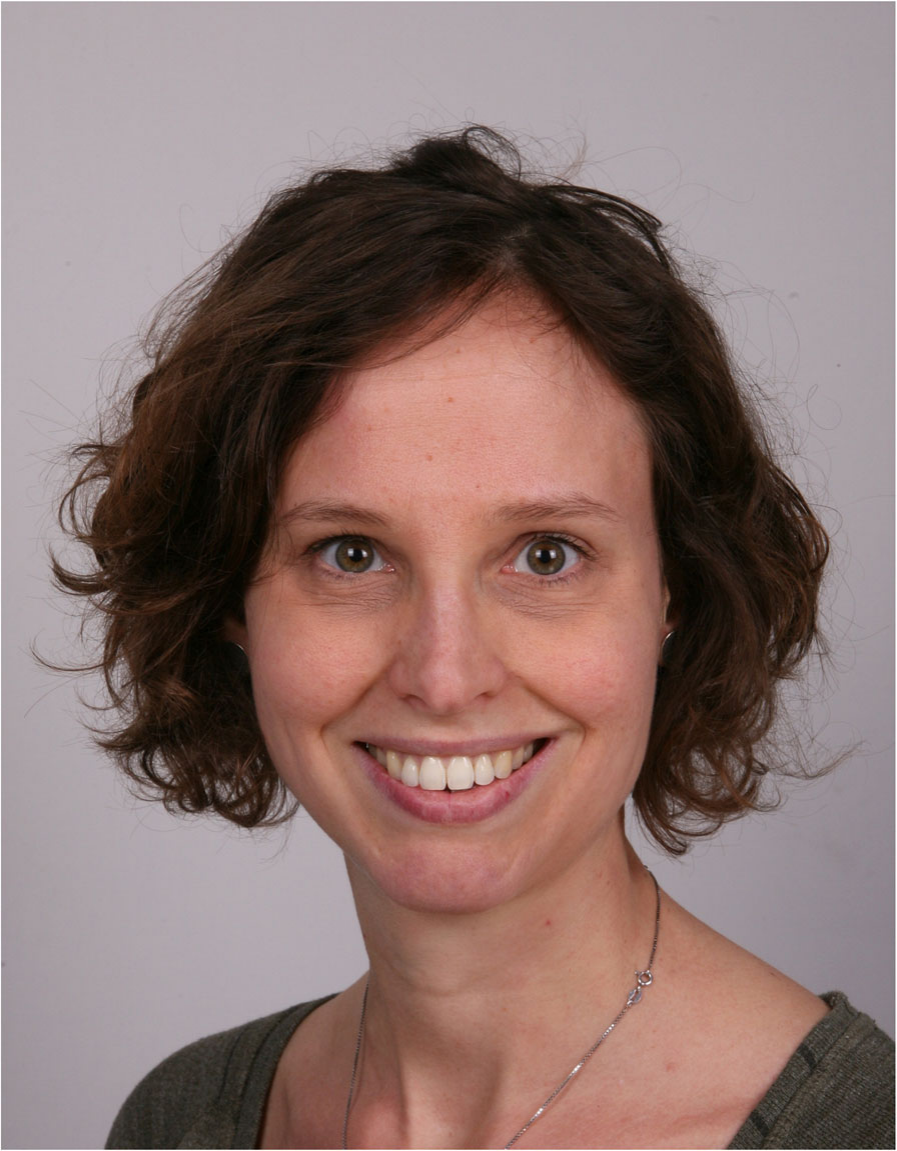
Annelieke Roest. Dr. Annelieke Roest works as an Assistant Professor at the University of Groningen, the Netherlands. Her research focuses on anxiety, including the epidemiology and treatment of anxiety disorders, as well as the association between anxiety and depression and somatic diseases

**Fig. 10:**
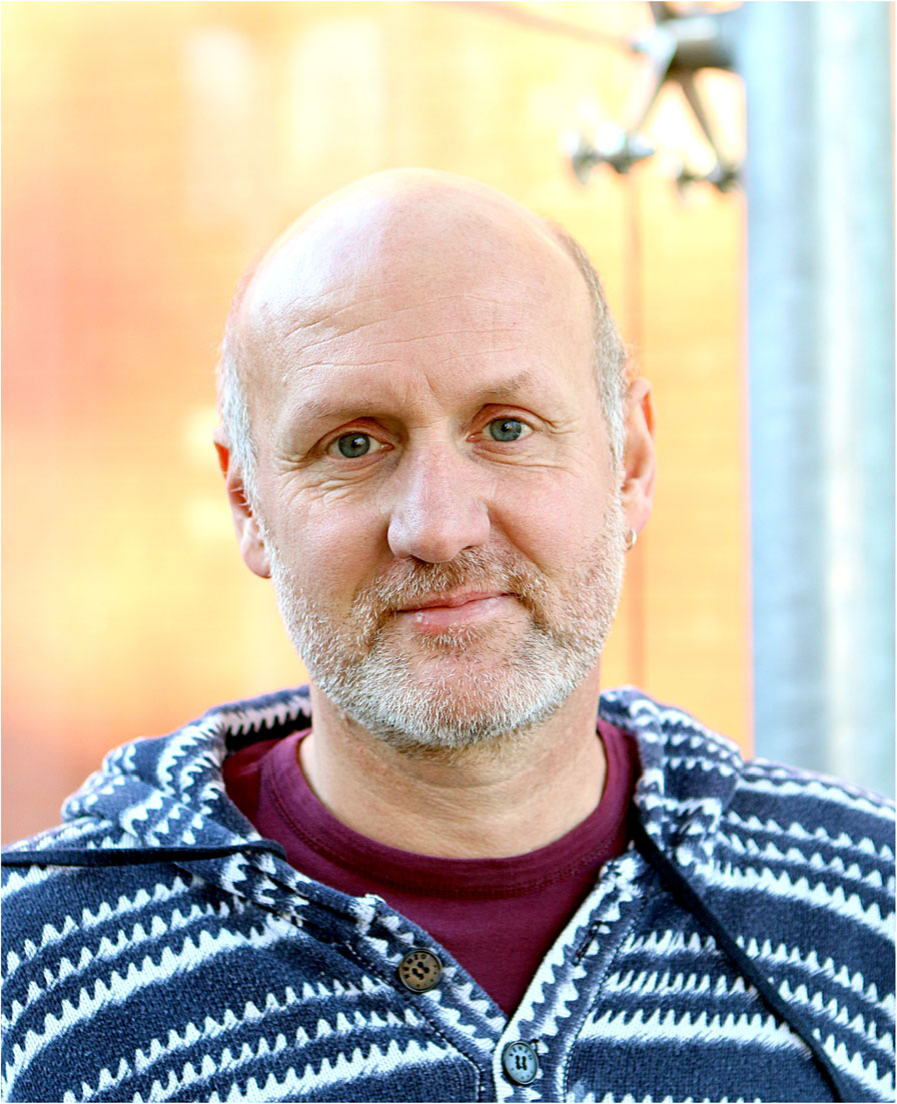
Peter de Jonge. Dr. Peter de Jonge is Professor and Head of Department of Developmental Psychology at the University of Groningen, the Netherlands. He is programme leader of the Interdisciplinary Center Psychopathology and Emotion Regulation (www.icpe.nl)

In the ICD-11, the classification of anxiety disorders has been simplified and brought into better agreement with an evidence-based Hierarchical Taxonomy of Psychopathology (HiTOP) [56]. This model proposes several higher-order dimensions, including internalizing and externalizing dimensions, and aims to give an aetiological account of mental disorders. The internalizing dimension is proposed to consist of several subdomains, including fear (e.g. phobia) and distress (e.g. generalized anxiety disorder (GAD), major depressive disorder). The ICD-10 grouped most anxiety disorders and OCD into the heterogeneous grouping of ‘neurotic, stress-related, and somatoform disorders’ (F40–F48), and also split anxiety disorders into ‘phobic anxiety disorders’ and ‘other anxiety disorders’ (GAD, mixed anxiety disorders, and panic disorder). While this split may have some face validity, factor analytic models generally find that panic disorder clusters with phobic disorders into the fear subdomain of internalizing disorders [56].

A new category of ‘anxiety or fear-related disorders’, which contains all anxiety disorders, including two (separation anxiety disorder and selective mutism) that were previously classed with childhood disorders, has been introduced in the ICD-11. Since anxiety disorders have similar symptoms (i.e. sympathetic arousal and avoidance), the ICD-11 emphasizes the focus of apprehension (e.g. fear of negative evaluation by others in the case of social anxiety disorder) as the basis for diagnostic differentiation between anxiety disorders [57]. Hierarchical exclusion rules, which often precluded explicit diagnosis of an anxiety disorder, particularly in individuals with mood disorders, have also been removed in the ICD-11.

OCD has been placed into its own grouping of ‘obsessive–compulsive or related disorders’. This grouping also includes several new disorders such as hoarding disorder and body-focused repetitive behaviour disorders (broadened from the ICD-10 diagnosis of trichotillomania). Hypochondriasis has also been moved from the category of ‘somatoform disorders’ into that of ‘obsessive–compulsive or related disorders’.

The ICD-11 has maintained the a priori split between mood and anxiety disorders, despite empirical findings that generally show that GAD is more closely related to major depressive disorder than it is to the other anxiety disorders. However, by providing anxiety disorders their own grouping, including separation anxiety disorder with the other anxiety disorders, and removing the artificial split between phobic and other anxiety disorders, the structure of the ICD-11 more closely approximates evidence-based models of the structure of psychopathology. Furthermore, such categorization brings the ICD-11 into closer agreement with the DSM-5.

Additionally, the ICD-11 closely resembles the DSM-5 in its disorder descriptions. Particularly, a requirement that the disorder should result in significant distress or impairment has been added to the description of all anxiety disorders. For agoraphobia, social anxiety disorder, specific phobia, and GAD, a specification that symptoms must persist for at least several months has also been added. Moreover, the conceptual core of several disorders, particularly panic disorder, GAD, and OCD, has been updated to reflect current beliefs about these disorders. With regard to panic disorder, the ICD-10 exclusively focused on the presence of unexpected panic attacks; however, in the ICD-11, persistent concerns about these panic attacks and/or attempts to avoid the recurrence of panic attacks are also considered essential and impairing features of panic disorder. In the ICD-10, GAD was conceptualized as free-floating worry that does not predominate in any particular environmental circumstance. While the ICD-11 maintains free-floating anxiety as a possible symptom, excessive worry focused on multiple everyday events is also recognized as a possible core symptom of GAD. Finally, the ICD-10 defined compulsions by their putative function (to prevent a feared event), while the ICD-11 has a broader definition, including all repetitive behaviors that an individual feels driven to perform in response to an obsession, according to rigid rules, or to achieve a sense of ‘completeness’. The ICD-11 also includes repetitive mental acts as compulsions, while the ICD-10 only focused on overt behaviour.

In sum, several of the changes in the ICD-11, both at the level of classification and at the level of disorder descriptions, result in greater agreement with the DSM-5 and with empirical evidence on comorbidity. Furthermore, the addition of impairment and duration criteria provides more guidance to distinguish disorder from normality; these changes, along with the updates to disorder descriptions, are likely to align the ICD-11 more closely with clinical practice.

## Disorders specifically associated with stress

### Andreas Maercker (Fig. 11) and Chris R. Brewin (Fig. 12)

The acknowledgment of stress as an external source of mental disorders is still relatively new in psychiatric nosology despite recognition that almost all mental disorders, to a greater or lesser degree, are shaped by it. For instance, psychosis tends to have much milder symptoms or even remain in a remission phase at low levels of external stress [58]. The ICD-11 goes further in recognizing this by including a new grouping of ‘disorders specifically associated with stress’ that identifies disorders in which external stress is a necessary and prominent causal factor. The grouping is parallel to ‘trauma and stress-related disorders’ in the DSM-5. However, the omission of the psychologically important but overused term ‘trauma’ in the ICD-11 grouping title was deliberate. The WHO Working Group decided that it was preferable to employ the term ‘stress’ so as to reduce the tendency to brand someone seeking professional help as psychologically ‘traumatized’.

**Fig. 11:**
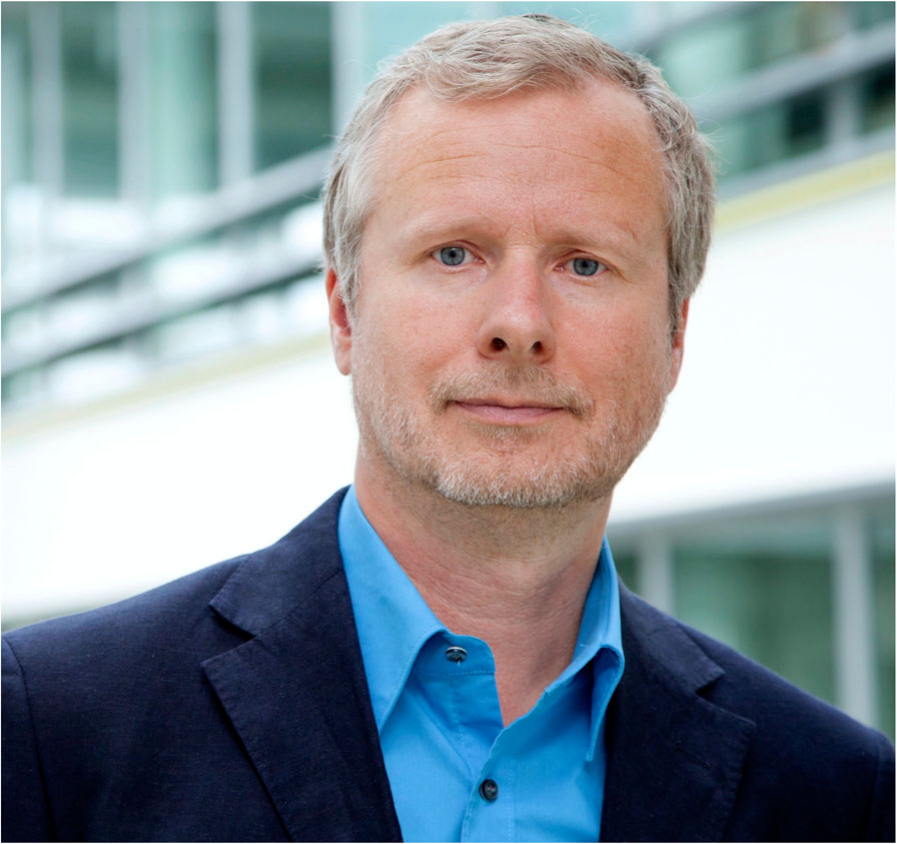
Andreas Maercker. Dr. Andreas Maercker is Professor and Chair of the Division Psychopathology and Clinical Intervention at the University of Zurich, Switzerland. His research interests include PTSD and its sibling disorders as well as cultural clinical psychology. He chaired the ICD-11 Working Group on Stress-related Disorders

**Fig. 12:**
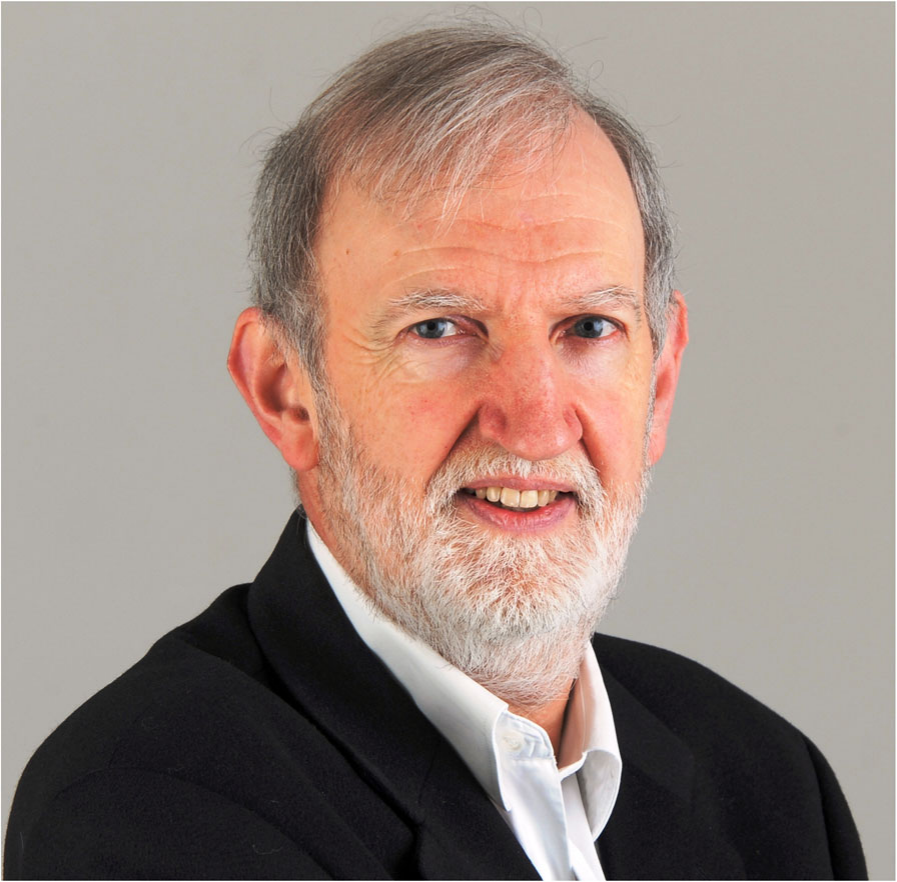
Chris Brewin. Dr. Chris Brewin, FAcSS, FMedSci, FBA, is Emeritus Professor of Clinical Psychology at University College London and a former consultant clinical psychologist at the Traumatic Stress Clinic, part of Camden & Islington NHS Foundation Trust

Moreover, the ICD and the DSM diverge significantly in their description of stress-related disorders — for the ICD-11, these are post-traumatic stress disorder (PTSD), complex PTSD, prolonged grief disorder, and adjustment disorder (described below), whereas for the DSM-5 they are PTSD, acute stress disorder, and adjustment disorder. (The respective groupings in both manuals also include similar desciptions of the childhood disorders of reactive attachment disorder and disinhibited social engagement disorder, which are not further discussed here.) The four new formulations of diagnoses within the ICD-11 grouping of disorders specifically associated with stress have generated a number of controversies.

#### PTSD

The ICD-11 has substantially simplifed the description of PTSD, defining it in terms of three core symptoms that most clearly distinguish PTSD from other disorders, namely re-experiencing the traumatic event or events in the present, deliberate avoidance of reminders, and a sense of ongoing threat. Furthermore, the symptoms must persist for at least several weeks and cause significant impairment in functioning. In contrast, PTSD is the most complex disorder in the DSM-5, with 20 symptoms organized into four symptom clusters. The intention of the DSM-5 was to capture the full scope of chronic post-traumatic phenotypes [59]. However, recent research has demonstrated that the data fit the simpler factor structure of the ICD-11 better than they do that of the DSM-5 and that, as intended, when using the ICD-11 definition there is a reduction in the degree of comorbidity with major depression [60].

#### Complex PTSD

It has been argued for many years that chronic or repeated trauma leads to a more severe form of PTSD. The ICD-10 contained a partly overlapping predecessor diagnosis of ‘enduring personality change after catastrophic experiences’, which had very rarely been used in clinical practice and research [61]. The ICD-11 defines complex PTSD as consisting of the three core PTSD symptoms described above accompanied by problems in affect regulation, negative self-beliefs, and relationship difficulties [62]. Chronic or repeated trauma is a risk factor rather than a requirement for the diagnosis, which is based on the symptom presentation. The ICD-11 definition nevertheless provides examples of experiences, such as torture, slavery, genocide campaigns, prolonged domestic violence, and repeated childhood sexual or physical abuse, that may be associated with the diagnosis.

Long-standing proposals to distinguish this disorder from PTSD were rejected in the DSM-5. However, empirical research using techniques such as latent class analysis and latent profile analysis has supported the distinction between PTSD and complex PTSD as well as the association between complex PTSD and trauma in childhood [60].

#### Prolonged grief disorder

The inclusion of this disorder in the ICD-11 followed careful consideration of the boundaries between normal and atypically severe grief as well as cultural/religious influences on the grieving processes. The evidence was judged sufficient to introduce a formal diagnosis for the minority of grieving individuals who may need professional services to overcome persistent and severe mourning [63]. The disorder is characterized by a pervasive longing for or persistent preoccupation with the deceased, accompanied by intense emotional pain. It can only be diagnosed if the symptoms persist beyond a period of 6 months — or longer if extended periods of acute grief are culturally normative for that individual [64]. In contrast to PTSD, where intrusive memories are generally characterized by fear or horror, preoccupation and longing often involve positive memories about the lost loved one. In the DSM-5, the evidence was only considered sufficient to include a somewhat differently defined ‘condition for further study’ involving symptoms lasting for at least 12 months after a death, termed ‘persistent complex bereavement disorder’.

#### Adjustment disorder

This frequently used but ill-defined diagnosis has been reformulated more precisely in the ICD-11 [65]. It is characterized by the presence of two symptoms, namely preoccupation with the stressors and indications of failure to adapt such as sleep or concentration problems [66]. It can be assigned some days after the stressor has impacted on the person, and it is typically expected to resolve within 6 months unless the stressor persists for a longer duration. Adjustment disorder is not a trivial condition — if it goes undetected and untreated it may lead to more severe mental disorders or an elevated risk of suicide [67, 68].

The four diagnoses presented here are intended to fulfill the aim of the ICD-11 to provide clear, simple diagnoses that meet the needs of clinicians worldwide. A field study conducted by the WHO with international practitioners found that the new distinction between PTSD and complex PTSD was easily applied but that there was some difficulty in applying the narrower definition of PTSD [69]. The forthcoming Clinical Descriptions and Diagnostic Guidelines will better educate clinicians in how to apply the descriptions of the required symptoms [20]. The more specific formulations are also likely to be beneficial to researchers who can now identify much more homogeneous groups of patients, assisting the search for biological markers. Comparisons with the DSM-5 will enable key diagnostic assumptions to be tested. The empirical data generated by the new diagnoses promise to yield a much greater understanding of how to recognize and treat these disorders in different settings worldwide [70–72].

## The classification of feeding and eating disorders

### Kathleen M. Pike (Fig. 13) and Carlos M. Grilo (Fig. 14)

The conceptual core of the ‘feeding and eating disorders’ (FED) grouping of disorders involves abnormal eating or feeding behaviors that are not better accounted for by other health conditions and are neither developmentally appropriate nor culturally sanctioned [73]. The ICD-11 classification of FEDs, guided by the principles of enhancing clinical utility and global relevance, includes changes supported by an evidence base accumulated during the more than 25 years since the ICD-10 was published and also supported by field trials [74]. This grouping combines feeding disorders and eating disorders, representing the integration of two previously distinct sections, a decision that parallels changes in the DSM-5 [5, 75].

**Fig. 13:**
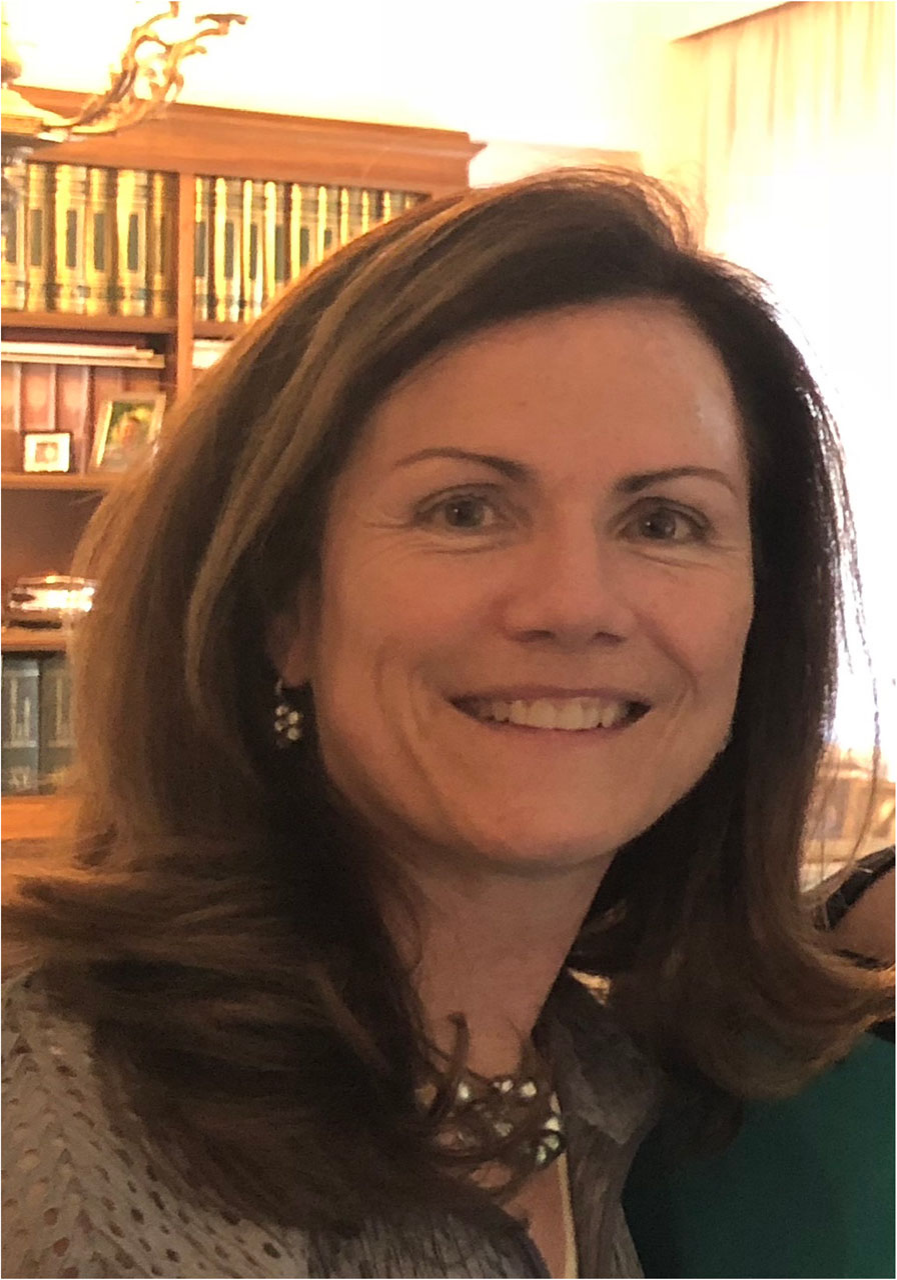
Kathleen M. Pike. Dr. Kathleen M. Pike is a clinical psychologist and Professor of Psychology in Psychiatry and Epidemiology at Columbia University Irving Medical Center. She serves as Director of the Global Mental Health WHO Collaborating Centre and Chair of the Faculty Steering Committee for the Global Mental Health Programs at Columbia. She was a member of the WHO ICD-11 Feeding and Eating Disorders Group, ICD-11 Field Studies Coordination Group, and the DSM-5 Cultural Consultation Group

**Fig. 14:**
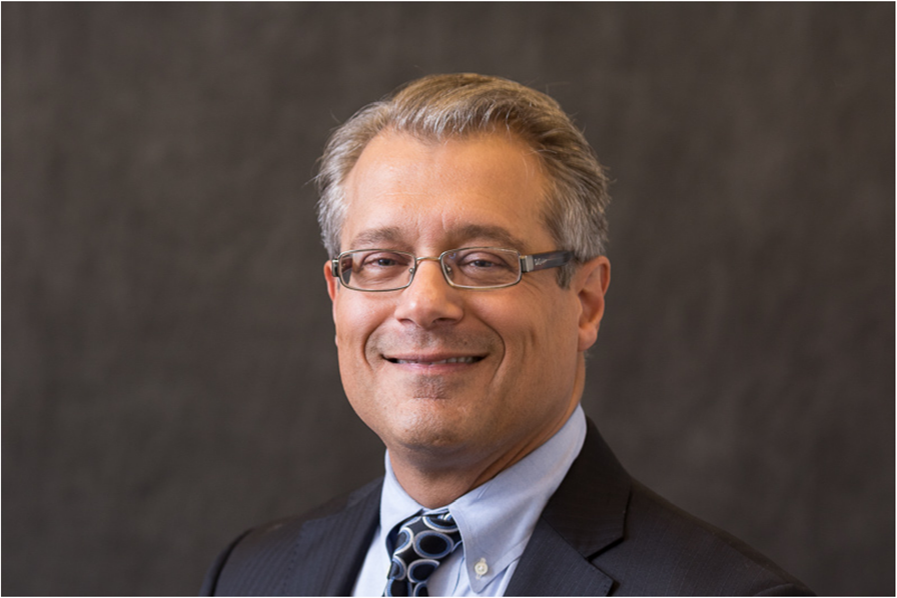
Carlos M. Grilo. Dr. Carlos M. Grilo, a clinical psychologist, is Professor of Psychiatry at the Yale University School of Medicine and Professor of Psychology at Yale University. He is the Founding Director of the Yale Program for Obesity, Weight, and Eating Research (POWER) and has, as Principal Investigator, been fully and continuously funded by the National Institutes of Health for 25 years. He previously served as Director of Psychology at the Yale Psychiatric Institute and as Director of Training in Clinical and Community Psychology at the Yale School of Medicine

Feeding disorders include a set of conditions characterized by restricted or limited intake (avoidant restrictive food intake disorder; ARFID), behavioral disturbances such as eating of non-edible substances (pica), or voluntary regurgitation of foods (rumination–regurgitation disorder) in the absence of shape/weight concerns. In contrast, eating disorders (anorexia nervosa (AN), bulimia nervosa (BN), and binge-eating disorder (BED)) are characterized by disturbances in eating behaviors accompanied by preoccupation with food, body weight, or shape in distinct patterns for each diagnosis.

Bringing together FEDs in a single grouping represents an expanded appreciation for the clinical significance of feeding problems during infancy and childhood [75, 76]. The guidelines for pica and rumination–regurgitation disorder have not changed substantially from the ICD-10. ARFID, which is characterized by restricted or limited food intake but not a disturbance in body image, represents a new diagnosis, though the feeding disturbances associated with ARFID have long been recognized by clinicians. We have limited research on the etiology, prognosis, natural outcome, or treatment of ARFID. The inclusion of ARFID in the ICD-11 should provide greater guidance regarding the operationalization of a working diagnosis, which we expect will stimulate attention and research that will advance understanding and treatment.

Among the eating disorders, the core conceptual characterization of AN and BN remain consistent with the ICD-10. However, detailed specifications regarding the essential features of these two disorders were revised based on updated empirical data and clinical practice. These revisions had the goal of enhancing the clinical utility of the diagnoses and reducing the use of ‘atypical’, ‘other specified’, or ‘unspecified’ diagnostic categories in the ICD-10, which have limited clinical utility or informational value.

For AN in the ICD-11, the hallmark feature remains, a low body weight for height, age, and developmental stage that is not better accounted for by another condition. The ICD-11 provides general guidelines for defining low weight. Specifically, it provides the commonly used guidelines of a body mass index (BMI) of less than 18.5 kg/m^2^ in adults and a BMI-for-age under fifth percentile in children/adolescents for diagnosing low weight. However, the ICD-11 guidelines indicate that these thresholds should be used as general points of reference but are not required thresholds for AN. This is important given cultural and individual variations, and thus allows AN to be diagnosed at higher weights in some circumstances. In addition, the essential features explicitly state that “*rapid weight loss (e.g., more than 20% of total body weight within 6 months) may replace the low body weight guideline as long as other diagnostic requirements are met*.”

Another feature added to the diagnosis of AN is the qualifier for the severity of underweight status, which reflects findings indicating that severe underweight status may have important prognostic implications in terms of increased risk of other health complications, increased mortality risk, and overall poorer outcome in adults [77–80]. The ICD-11 specifier of severity of underweight for AN in some ways roughly parallels a change in the DSM-5 in which AN severity is based on BMI. To date, research has provided limited support for the DSM-5 low BMI specifier ratings across both European [81–84] and US [85, 86] samples. However, one study found that lower BMI categories were significantly related to indicators of need for greater services such as number of hospitalizations [85].

The ICD-11 qualifiers for AN related to low body weight include ‘significantly low body weight’ and ‘dangerously low body weight’. The third qualifier related to weight status is ‘anorexia nervosa in recovery with normal body weight’. The ICD-11 included this qualifier to avoid the previously existing conundrum that individuals who achieved weight restoration while in treatment would no longer meet diagnostic thresholds for AN despite continuing to have all the other symptoms of AN and not being sufficiently recovered to maintain the weight gain without continued treatment. The technical inability to diagnose someone with AN at such a point in care was particularly problematic in certain contexts in which a diagnosis is essential for receiving certain levels of care based on health systems and policies. The proposed qualifier provides the elegant solution of identifying the weight gain while still retaining the diagnosis of AN. As provided by this qualifier, the diagnosis of AN is retained “*until a full and lasting recovery is achieved, as indicated by the maintenance of a healthy weight and the cessation of behaviours aimed at reducing body weight, independent of the provision of treatment (e.g., for at least 1 year after intensive treatment is withdrawn)*”. In contrast to the DSM-5, the ICD-11 retains the qualifiers of ‘restricting pattern’ versus ‘binge–purge pattern’ of compensatory weight-related behaviors.

In the case of BN, the conceptual core remains centred around binge-eating behaviour coupled with regular and significant weight-compensatory behaviors. Two key changes regarding the clinical assessment of binge-eating behaviour are noteworthy. First, the frequency threshold of binge eating for BN was reduced to once a week or more over a period of at least 1 month. The lowered frequency/duration stipulations represent both empirical data and prioritization of clinical utility. Data assessing ‘subthreshold’ BN indicate that individuals who binge eat at a frequency of once weekly are comparable to those individuals who do so more frequently [87]. The shortened duration reflects the clinical reality that access to care is limited in many parts of the world, and if someone presents with all the features of BN for a duration of 1 month, they should receive clinical care without further delay.

In addition to the changes in specifications to AN and BN, the most significant addition to the eating disorders grouping was the inclusion of BED as a separate category. BED is a disturbance characterized by recurrent binge eating, associated with significant distress, in the absence of inappropriate weight-compensatory behaviors. The addition of BED as a formal diagnosis, which parallels changes in the DSM-5, follows two decades of research on the clinical significance and validity of this diagnostic construct [88], which had previously been described as a research category in the Appendix of the DSM-IV. Emerging research worldwide has indicated the prevalence of BED relative to other eating disorders and established the clinical and public health significance of this diagnostic construct [89, 90].

Both BN and BED are based on the occurrence of binge eating, and the ICD-11 also incorporates changes regarding the definition of what constitutes an episode of binge eating. In the ICD-11, binge eating is defined as a time when an “*individual eats notably more and/or differently than usual and feels unable to stop eating or limit the type or amount of food eaten. Other characteristics of binge-eating episodes may include eating alone because of embarrassment, eating foods that are not part of the individual’s regular diet, eating large amounts of food in spite of not feeling hungry, and eating faster than usual*” [74]. These other features reflecting a general loss of control or a difference in the eating behaviors both resonate with clinicians and patients and parallel the behavioral indicators for establishing binge eating in the DSM-IV/DSM-5, which have received empirical support [91]. Also notable is that the ICD-11 guidelines have eliminated the requirement that binge-eating episodes be defined by an objectively unusually large amount of food. This change, which is at odds with the DSM-5, is consistent with increasing evidence suggesting that the subjective experience (most notably a sense of loss of control) is more important that the quantity eaten during those episodes [92, 93].

In both BN and BED, there is marked distress about the pattern of binge eating or significant impairment in personal, family, social, educational, occupational, or other important areas of functioning. Distress associated specifically with binge eating has been shown to be an important discriminatory feature [94]. Interestingly, while the ICD-11 requires marked distress for both BN and BED, the DSM-5 does so for BED but not for BN, perhaps due to the clinical assumption that the extreme weight-compensatory behaviors undoubtedly reflect distress. Finally, for both AN and BN, a disturbance in body image is required, whereas for BED the ICD-11 notes that, while overvaluation of shape/weight is commonly present, it is not required; this is at odds with the DSM-5 for BED but is consistent with empirical data [20, 95].

The new ICD-11 guidelines for FED, which follow empirical advances and received support in the field trial [74], should facilitate clinical practice worldwide. As noted above, several of the major changes and additions have broadened the diagnostic guidance in a balanced fashion to better capture clinical realties and reduce ‘technicalities’ that do not appear to be clinically or empirically meaningful (e.g. examples of what constitutes binge eating, frequency/duration stipulations, definition of abnormally low weight) but that can delay clinical care. Although critics might voice concerns that some of the changes involving broadening of certain features (i.e. removing arbitrary symptom counts and duration cut-points not supported by research) as compared to the DSM-5 might result in over-diagnosis, a recent large-scale epidemiological study in the US did not provide support for such concerns [96]. We believe that the ICD-11 guidelines effectively capture the diverse clinical realities across the developmental life course and around the world, and we anticipate that the changes made in the ICD-11 guidelines for FEDs will aid better diagnosis and treatment.

## Disorders of addictive behaviour

### Naomi A Fineberg (Fig. 15)

The ICD-11 revision heralds a welcome sea change in the clinical conceptualization of addictive behaviour. First and foremost, by introducing the new section ‘Disorders due to substance use or addictive behaviours’, the ICD-11 brings together substance use disorders with disorders of addictive behaviour under one conceptual framework. In this respect, it aligns with and expands upon similar changes made in the DSM-5 [5]; the central role of addiction, as a trans-diagnostic process underpinning a broad range of harmful behaviors, is prioritized and its key behavioral constituents are defined as impaired control, precedence over other interests and activities, and continuation or escalation of the behaviour despite negative consequences.

**Fig. 15:**
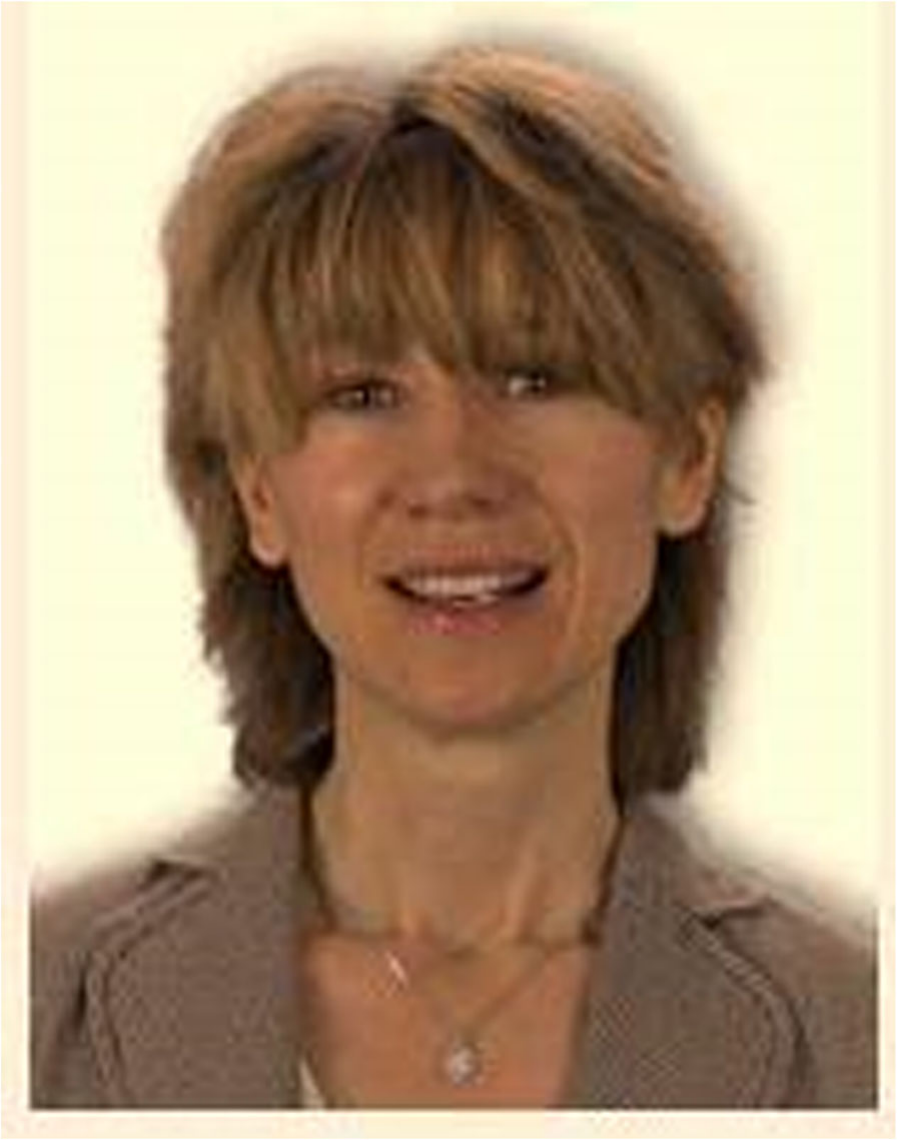
Naomi A Fineberg. Dr. Naomi A Fineberg is a consultant psychiatrist and Professor of Psychiatry at the University of Hertfordshire, and Hertfordshire Partnership University NHS Foundation Trust, UK. She specializes in the research and treatment of obsessive–compulsive and related disorders. She chairs the World Psychiatric Association Scientific Section for Anxiety and Obsessive–Compulsive Disorders and an EU COST Action for the Study of Problematic Internet Usage

Second, the ICD-11 recognizes, for the first time, a group of ‘disorders due to addictive behaviours’. These are defined as clinically significant syndromes associated with distress or dysfunction that develop as a result of repetitive rewarding behaviors other than the use of dependence-producing substances. They include gambling disorder (previously classified with habit and impulse disorders) and gaming disorder (a new diagnosis), both of which may involve online or offline behaviour. In the ICD-11, the definition of gambling disorder has been adjusted in line within the addiction framework.

Third, gaming disorder is accepted as a diagnosis for the first time (see below), defined by persistent or recurrent addictive online or offline gaming of such severity so as to cause significant impairment in personal, family, social, educational, occupational, or other important areas of functioning. Fourth, the Internet is included as a diagnostic specifier, to the extent that gambling disorder can be separately diagnosed as being predominantly offline or online. Fifth, by creating residual categories (‘other specified’ or ‘unspecified disorders of addictive behaviour’) attention is given to individuals not meeting the diagnostic criteria for an existing disorder, who nevertheless experience significant distress or impairment associated with a range of otherwise neglected addictive behaviors that might include shopping, stealing, pornography viewing, web-streaming, social media use, and other behaviors, for which there is as yet insufficient evidence to justify classification as a diagnosis. Finally, as not all forms of gambling or gaming meet the threshold for a diagnosable condition, definitions for ‘hazardous gaming’ and ‘hazardous gambling or betting’ have additionally been included as alternatives to diagnostic entities so that public health may be promoted.

The rationale for these recommendations is derived from in-depth scientific analysis coupled with multi-professional clinical and public health experience, involving experts from over 25 countries; changes from the ICD-10 were debated in a series of workgroup meetings [97]. Indeed, discussion in the scientific literature has continued as emerging data suggests that, alongside the phenomenological and psychobiological overlaps between gambling and gaming disorders and disorders of substance addiction [98, 99], including high levels of clinical comorbidity, overlaps are also to be found with certain impulse control disorders, such as kleptomania and compulsive sexual behaviour disorder, and obsessive–compulsive or related disorders such as trichotillomania and excoriation disorder [100, 101]. Thus, as research grows and our understanding of these and other putative addictive disorders crystallizes, further revisions to some of these ICD sections may arise.

In its deliberations, the ICD-11 focused on clinical and public health utility, with an explicit aim of improving primary care in non-specialist settings [18]. The new classification is thus expected to raise awareness of these otherwise overlooked disorders of behavioral addiction among clinicians and the public and facilitate the development of clinical and public health interventions. The new classification will also invigorate research into the role of the Internet as both a conduit and a potential moderator of addictive behaviors.

The inclusion of gaming disorder in the ICD-11 forms a basis for the development and implementation of standardized diagnostic interviews and symptom measures, potentially leading to the discovery of effective interventions. However, this has generated a lively debate, with some authors expressing concern that the scientific basis for gaming disorder is currently too weak or that non-problematic gamers could be stigmatized by its inclusion [102, 103]. A series of commentaries published in the last year (reviewed in [98]) has largely favored the inclusion of gaming disorder in the ICD-11. Evidence demonstrating the negative effects of pathological gaming in multiple psychosocial domains [104] played an influential role.

While it is laudable to consider the unwanted effect of stigmatization when a diagnosis is newly introduced [105], this argument must be balanced against clinical and public health needs [97]. Treatment demand worldwide and the significant distress, functional impairment, and suffering encountered by those experiencing disorders of behavioral addiction, such as gambling and gaming disorder, underlies the pressing need for their adoption by the ICD-11. By classifying these disorders within an addiction framework, the ICD-11 endorses the approach that addictive behaviour is not exclusively a medical problem, and that prevention and reduction of the associated health and social burden may be achieved by interventions inside and outside the health sector [1].

## Compulsive sexual behaviour disorder

### Peer Briken (Fig. 16)

The decision to include compulsive sexual behaviour disorder (CSBD) in the group of impulse control disorders in the ICD-11 marks a paradigm shift from a sexual and mental health perspective, especially considering that CSBD is now precisely described through diagnostic guidelines [2, 100]. Why consider this a shift in paradigm? The ICD-10 included the category ‘excessive sexual drive’, which, however, did not include a specific description of the symptoms but did reference ‘nymphomania’ and ‘satyriasis’. The diagnosis was placed in the grouping of sexual dysfunctions within the Mental and Behavioral Disorders chapter of the ICD-10 because it concerned sexual behaviour.

**Fig. 16:**
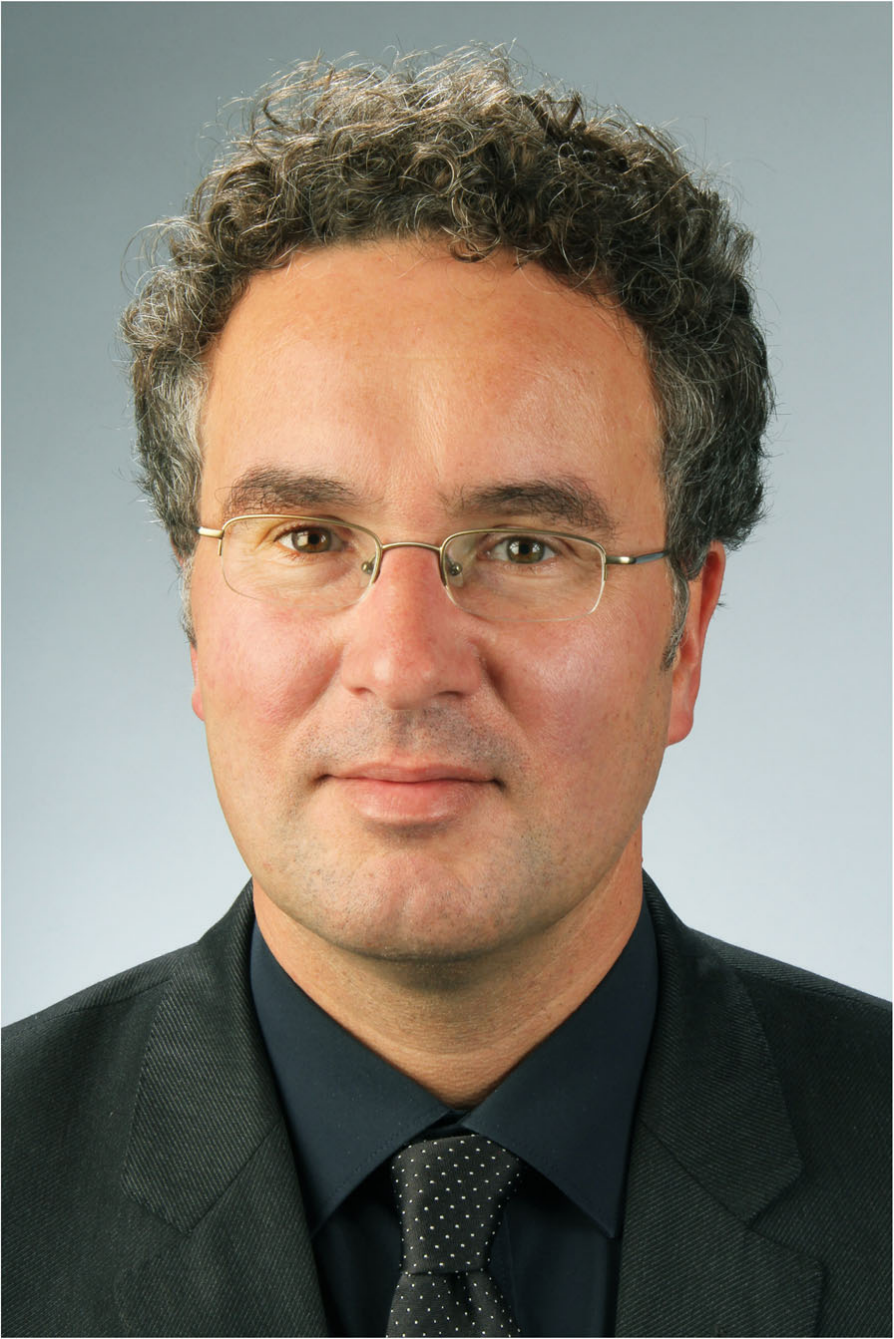
Peer Briken. Dr. Peer Briken, is Professor for Sex Research, Sexual Medicine, and Forensic Psychiatry and Director of the Institute for Sex Research, Sexual Medicine, and Forensic Psychiatry, University Medical Centre Hamburg-Eppendorf, Germany, since 2010. He was President of the German Society for Sex Research from 2010 to 2016. Since 2016, he is a member of the Independent Commission for the Inquiry of Sexual Abuse of Children in Germany. He is a consultant to the World Health Organization regarding the development of the ICD-11 classification of sexual disorders and sexual health

In contrast, the ICD-11 guidelines describe CSBD as characterized by “*a persistent pattern of failure to control intense, repetitive sexual impulses or urges resulting in repetitive sexual behaviour*” ([2], p. 109) over an extended period. In persons with this disorder, sexual behaviour has become a central focus of their life, with unsuccessful efforts to control or significantly reduce it as well as adverse consequences (e.g. repeated relationship disruption, occupational consequences, negative impact on health). The behaviour results in marked distress or significant impairment. The ICD-11 diagnostic guidelines largely avoid focusing on possible distal aetiological issues (e.g. traumatic sexual experiences) and the many contexts in which compulsive sexual behaviour occur (e.g. using sex as a coping strategy in response to negative emotions). This is advantageous because CSBD is an umbrella construct that likely has a variety of aetiologies not directly relevant to diagnostic description.

Although the word ‘compulsive’ is included in the name because that is a very common way of referring to this phenomenon in the literature, sexual behaviour in CSBD is not considered a true compulsion that occurs in relation to intrusive, unwanted and typically anxiety-provoking thoughts (obsessions) as in OCD. Rather, CSBD is a repetitive, typically initially rewarding behaviour pattern that the person feels unable to control, which appears to have both impulsive and compulsive elements [106]. Early in the development of the behaviour pattern, impulsivity and positive reinforcement (pleasure) are the most important elements. Later in the course of the disorder, compulsive aspects and negative reinforcement (e.g. temporary improvement of negative mood) are likely to become increasingly important in sustaining the behaviors [107]. The most thoroughly investigated theoretical model on the interplay between excitatory and inhibitory influences on sexual behaviour is the dual control model [108], according to which CS BD could be a problem when sexual self-control is relatively low and sexual responsiveness/excitability is relatively high [109, 110]. Clinically, the lack of self-control is often subjectively experienced as urgency, while sex in CSBD may fulfill a variety of different functions. Treatment therefore focuses on improvement of sexual self-control as well as coping with the underlying emotional states and motives.

Available representative community data indicate that subjective difficulty in adequately controlling sexual behaviour is common [111], although such subjective difficulty alone is not sufficient for a diagnosis of CSDB. The ICD-11 guidelines indicate that CSBD *“should not be diagnosed based on distress related to moral judgements and disapproval about sexual impulses, urges, or behaviours that would otherwise not be considered indicative of psychopathology (e.g. a woman who believes that she should not have sexual impulses at all; a religious young man who believes that he should never masturbate; a person who is distressed about their homosexual attraction or behaviour).”* Available evidence suggests a prevalence of 10–12% for men and 7% for women for sexual compulsivity; however, the new CSBD guidelines are yet to be used in epidemiological studies (e.g. [111–113]).

How will this diagnostic category advance the field? From a sexual and mental health perspective, inclusion in the classification system suggests that CSBD can be treated, that mental health service systems should be capable of providing the treatment, and that this treatment should be covered by health insurance. Furthermore, only a disorder that is constructed with regards to valid and reliable guidelines can be researched accordingly. In the short time since the conceptualization of CSBD as an impulse control disorder has been put forward [100] there have been numerous new studies, with the focus of this research being significantly broadened by a range of new research groups. Research has investigated disorder constructs related to CSBD [114] and validation of instruments [115] as well as aetiological [106, 114] and therapeutic approaches [116–118]. The significance of impulsivity, compulsivity, and addiction is still the subject of ongoing studies and discussions in the constructs of CSBD and has not been conclusively clarified.

From a historical point of view, it should be noted that clinicians and scientists [119] described a forerunner diagnosis to CSBD more than 100 years ago. In German psychopathology, there has been a discussion about compulsive or dysregulated forms of sexual expression since the 1940s (overview in Giese [120]). The discussion became internationally prominent in the US following the publication, in 1983, of Carnes’ book Out of the Shadows: Understanding Sexual Addiction [121], which provided a conceptualization of the sexual addiction model and how 12-step self-help programmes could be helpful. Nevertheless, despite some popularization of this idea, it has also received significant criticism [122]. At a minimum, it must be acknowledged that, in some parts of the world, including North America, there is a risk that any conceptualization of non-mainstream sexuality as disordered will be seized upon in unintended, sex-negative ways based on religious attitudes hostile to sexuality and by religiously based therapies or self-help groups. This potentially creates a problem for a scientifically sound approach to CSBD.

In order to rule out inappropriate pathologization and protect against misuse, the guidelines state that “*distress that is entirely related to moral judgments and disapproval about sexual impulses, urges, or behaviours is not sufficient to meet this requirement*”. The guidelines further indicate that high levels of sexual interest and behaviour in the absence of impaired sexual self-control and distress or impairment, or during relatively brief periods, should not be diagnosed as CSBD. Similarly, high levels of sexual interest and behaviour in adolescents should not be labeled as CSBD, even if associated with distress.

It has sometimes been claimed (e.g. [123]) that the idea that sexual behaviour can be compulsive or resemble an addiction emerged as a product of conservative (e.g. religious) sexual norms, accompanied by new psychiatric diagnostic labels. However, in the case of CSBD this does not seem to be true. Rather, changes in social and environmental conditions (e.g. digital media) have led to changes in symptomatic expression, which in turn may suggest the need to alter diagnostic classifications. We live in a digital world where the availability of pornography and the possibilities of sexual interactions without prolonged relationships have changed the reasons why patients come into clinical practice.

Accordingly, in our clinic, there has not been a change in the numbers of patients who present for treatment, yet changes in the manifestation of this condition have been observed. For example, 20 years ago, most cases involved visits to peep shows or use of sex workers or telephone sex, whereas, currently, CSBD is primarily related to the use of digital media, most often with uncontrolled use of pornography sites or multiple sexual contacts via dating apps. Of note, when CSBD occurs in combination with a paraphilic disorder or criminal sexual behaviour it has a different profile.

In clinical practice, the new guidelines should help ensure that the threshold for diagnosis is not too low and thus running the risk of false positive diagnoses. On the other hand, it is important that the threshold emphasizes clearly pathological behaviour — not all individuals who claim they are ‘sex addicts’ automatically fulfill the diagnostic requirements for CSBD. Indeed, the ICD-11 aims to identify those individuals for whom CSBD is a serious problem and who should appropriately receive treatment.

## Gender identity-related diagnoses

### Peggy T. Cohen-Kettenis (Fig. 17)

Since the first publications of gender identity-related diagnoses in the ICD and the DSM [5], these diagnoses have undergone significant changes in conceptualization, terminology, and placement. In the ICD-10, the diagnoses were ‘gender identity disorder’ for children and ‘transsexualism’ for adolescents/adults. Conversely, the DSM-IV had one overarching diagnosis of ‘gender identity disorder’ for both children and adolescents/adults, albeit with different indicators for the two groups. In the ICD-11, the names of the diagnoses have been changed, reflecting a different conceptualization of the phenomenon; this was also the case in the DSM-5 to some extent. In the ICD-11, the diagnoses are ‘gender incongruence of childhood’ for pre-pubertal children and ‘gender incongruence of adolescence and adulthood’ for post-pubertal individuals. These names highlight the core of the condition, that is, incongruence between a person’s experienced gender or gender identity and the sex (usually) assigned at birth. The DSM-5 uses the term ‘gender dysphoria’ for all age groups.

**Fig. 17:**
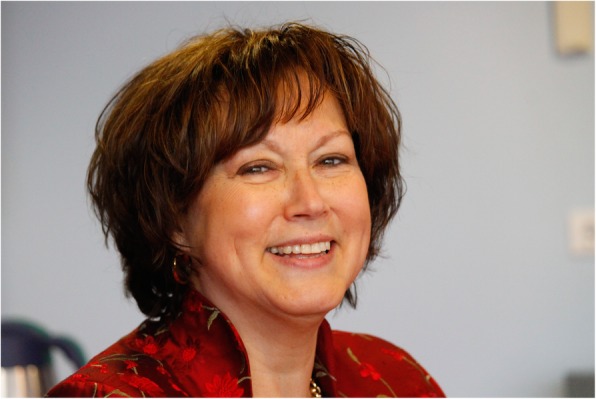
Peggy Cohen-Kettenis. Professor Peggy Cohen-Kettenis is Professor Emeritus of Medical Psychology at the Amsterdam UMC, Vrije Universiteit Amsterdam, the Netherlands, and was Director of the hospital’s Center of Expertise on Gender Dysphoria. She was a member of the World Professional Association for Transgender Health’s Standards of Care Committee and of the Task Force of the Endocrine Society Clinical Practice Guideline on the endocrine treatment of gender-dysphoric/gender-incongruent persons. She was Chair of APA’s DSM-5 GID subcommittee and member of the WHO ICD-11 Working Group on Sexual Disorders and Sexual Health

There has been much dispute on the question of whether gender-variant identities should be viewed as mental disorders or as variations within the normal range. Gender incongruence may or may not be associated with distress. In the DSM-5, clinically significant distress is a requirement for the diagnosis. Lesbian, gay, bisexual, and transgender advocacy groups have lobbied to remove gender dysphoria altogether from the DSM-5 as retention in this psychiatric classification system was viewed as stigmatizing. Despite this opposition, the American Psychiatric Association decided to retain the diagnosis. People may seek psychological care or gender-confirming treatment to alter physical sex characteristics (e.g. by hormone treatment and/or surgery) and support services need a billable diagnosis code. Removing gender dysphoria from the DSM was expected to create serious problems with access to care in many countries, which contributed considerably to the American Psychiatric Association’s decision to keep gender dysphoria in the DSM.

In the absence of distress, gender incongruence cannot be conceptualized as a mental disorder, yet people may need treatment. Although the prevalence of mental health problems is elevated among transgender people and distress (internally driven or as a result of outside forces) may be present, clinically significant distress does not seem to be inherent in gender incongruence [124, 125]. The ICD is a broader document than the DSM, as it encompasses mental disorders as well as other conditions needing treatment. The WHO, a United Nations agency, views health as a human right, creating a legal obligation for member states not only to ensure access to timely and affordable healthcare of good quality, but also to provide for the underlying determinants of health [126]. Stigma may be one such determinant, as there is evidence that it is associated with health problems [127, 128]. In many places, mental illness is stigmatized [129, 130] and stigma against transgender people is also highly prevalent [131, 132], creating a ‘double stigma’ burden for this community. In order to avoid this double stigma, the WHO, advised by the Working Group on the Classification of Sexual Disorders and Sexual Health, moved the gender identity-related diagnoses away from the mental disorders chapter and placed them in a new chapter entitled Conditions Related to Sexual Health in the ICD-11. This change allows for the diagnosis in a classification system of conditions that may need treatment, and therefore ensures access to care for transgender people without the additional burden of requiring a mental health disorder diagnosis.

There has been great concern that pre-pubertal children who only show gender-variant behaviour but have no gender identity-related problems would erroneously receive a diagnosis and be treated by clinicians and parents as future transgender adults [133]. By introducing an anatomic dysphoria requirement and lengthening the required duration of the gender incongruence, the ICD-11 diagnosis for children has been narrowed considerably. However, despite this tightening of requirements and the move away from the mental disorder chapter, there are still differences of opinion regarding the retention of gender incongruence of childhood in the ICD-11; some feel that even a non-psychiatric diagnosis can be harmful to children [134]. However, although pre-pubertal children do not need a diagnosis for medical treatment, they may need specialized supportive mental health services as well as family and social (for example, school) interventions. Retention helps to preserve access to care for a vulnerable and already stigmatized group [135].

Another major conceptual change for gender incongruence diagnoses in ICD-11 is a move away from binary thinking. Instead of the concepts of ‘male’ versus ‘female’ and ‘transsexual’ versus ‘non-transsexual’, the ICD-11 reflects evidence of considerable variance in gender and gender identity. As a result, people experiencing some form of gender incongruence who would previously be considered non-transsexual [135], and therefore not eligible for ‘classical’ gender affirming treatment (hormones first, followed by various types of surgery), may now be eligible for some form of medical treatment. Type and timing may differ from the ‘classical’ treatment.

The inclusion of gender incongruence diagnoses in the ICD-11 with the revised diagnostic guidelines and outside the mental health chapter will provide access to appropriate and non-stigmatizing care for those individuals with gender incongruence. It will also provide opportunities for the much-needed education of health professionals and facilitate the research and development of standards of care. The gender incongruence diagnoses may thus guide clinicians and family members in making decisions on clinical and non-clinical interventions.

## Conclusion

### Dan J. Stein and Geoffrey M. Reed

Overall, our view is that the various changes made in the Mental, Behavioral or Neurodevelopmental Disorders chapter of the ICD-11 contribute to a significant advancement in diagnostic classification as well as in mental health practice and research. However, the ways in which the ICD-11 as well as other important frameworks, including the DSM-5 and the Research Domain Criteria, have and have not advanced the field need to be further understood. In order to do so, two key issues, namely causality and thresholding, should be addressed.

Medical classifications that are based on etiology have important advantages; such a classification system itself provides clear targets for intervention. In the case of mental disorders, this is not possible because the etiology of mental disorders is not only multicausal but also incompletely understood. Importantly, the ICD-11 chapter on Mental, Behavioral or Neurodevelopmental Disorders incorporates the issues of etiology in a number of ways — certain groupings of disorders reflect common underlying mechanisms (e.g. disorders of addictive behaviour), certain diagnoses are based on specified causes (e.g. anxiety due to substance use or to a medical disorder), and certain causal factors can be classified (e.g. in the chapter on Factors Influencing Health Status and Contact with Health Services) but are not considered to be disorders. Continuous revisions of the nosology are important in ensuring that etiology is included to the maximal extent possible, and the ICD-11 chapter on Mental, Behavioral or Neurodevelopmental Disorders therefore represents a useful update.

However, the fact that the ICD-11 classification of mental disorders is not entirely based on causality raises some important considerations. First, there is not necessarily a close relationship between diagnosis, underlying neurobiological vulnerabilities, social determinants, and appropriate intervention. Second, there is likely to be some artefactual increase in comorbidity — multiple diagnoses of mental disorders in a particular individual may not indicate that a range of truly separate sets of underlying psychobiological processes are involved but rather may simply point to greater underlying dysfunction. Third, there is a temptation to reify psychiatric constructs, inappropriately regarding them as natural kinds [136]. All of these considerations point to the importance of regarding mental disorder classification systems as tentative, of emphasizing the need for careful individualized assessment, and of recognizing the need for a range of interventions to address the multiple causal factors at play in mental illness.

Medical classifications face the perennial problem of drawing thresholds among disorders as well as between such disorders and normality; this is a particularly fraught issue in mental disorders, where critics have raised concerns about inappropriate over-medicalization. The hope that advances in neuroscience would lead to biomarkers with sensitivity and specificity has not materialized. Importantly, given its focus on use in a wide range of settings, the WHO Department of Mental Health and Substance Abuse is providing diagnostic guidelines for the ICD-11 chapter on Mental, Behavioral or Neurodevelopmental Disorders that allow flexible use of clinical judgment, rather than focusing on narrow stipulations of symptom counts or duration that may improve reliability but not necessarily validity. These ICD-11 diagnostic guidelines for use in clinical settings have, however, undergone rigorous field testing; changes in the diagnostic guidelines from ICD-10 to ICD-11 are not simply arbitrary or capricious, but rather reflect the outcome of ongoing developments in nosological science.

Nevertheless, the fact that the ICD-11 chapter relies on clinical guidelines rather than on specific cut-points to draw boundaries, again raises a number of important considerations. First, the ICD-11 diagnostic guidelines place a great deal of emphasis on the ‘clinical criterion’ — the distress and impairment that are associated with symptoms. Second, although dimensional constructs have been included in a number of places in the ICD-11, this remains a largely categorical system that does not incorporate fine-tuned assessment of symptom variations. Third, there will again be a temptation to reify the particular boundaries set by the classification system, even where this is explicitly not intended, inappropriately overlooking the burden of disease associated with subclinical presentations or inappropriately underestimating the resilience of those who meet clinical thresholds. These considerations again point to the tentative nature of mental disorders classifications, to the need for supplementary individualized assessment, and to the importance of non-reductionistic conceptual approaches.

In summary, our view of psychiatric classification in general, and of the ICD-11 in particular, is one that avoids the overly optimistic view that nosology can or should resolve all the key debates in the field, and one that steers clear of the overly pessimistic view that the classification of mental disorders is inherently deeply and necessarily flawed. Global mental health is intimately linked with the sustainable goals for development [137], and the ICD-11 provides an important tool for these endeavors. In particular, its focus on global applicability and clinical utility in non-specialized primary care settings will maximize the likelihood that it will be adopted by mental health professionals and administrators as well as enhance its application in non-specialist settings and its usefulness for scaling up evidence-based interventions. The new mental disorders classification in ICD-11 and its accompanying diagnostic guidelines therefore represent an important, albeit iterative, advance for the field.
